# Anxiety-like behavior during protracted morphine withdrawal is driven by gut microbial dysbiosis and attenuated with probiotic treatment

**DOI:** 10.1080/19490976.2025.2517838

**Published:** 2025-06-15

**Authors:** Mark Oppenheimer, Junyi Tao, Shamsudheen Moidunny, Sabita Roy

**Affiliations:** a Neuroscience Graduate Program, University of Miami Miller School of Medicine, Miami, USA; b Department of Surgery, University of Miami Miller School of Medicine, Miami, USA

**Keywords:** Opioid withdrawal, anxiety, gut-brain axis, amygdala, probiotics, serotonin

## Abstract

The development of anxiety during protracted opioid withdrawal heightens the risk of relapse into the cycle of addiction. Understanding the mechanisms driving anxiety during opioid withdrawal could facilitate the development of therapeutics to prevent negative affect and promote continued abstinence. Our lab has previously established the gut microbiome as a driver of various side effects of opioid use, including analgesic tolerance and somatic withdrawal symptoms. We therefore hypothesized that the gut microbiome contributes to the development of anxiety-like behavior during protracted opioid withdrawal. In this study, we first established a mouse model of protracted morphine withdrawal, characterized by anxiety-like behavior and gut microbial dysbiosis. Next, we used fecal microbiota transplantation (FMT) to show that gut dysbiosis alone is sufficient to induce anxiety-like behavior. We further demonstrated that probiotic therapy during morphine withdrawal attenuated the onset of anxiety-like behavior, highlighting its therapeutic potential. Lastly, we examined transcriptional changes in the amygdala of morphine-withdrawn mice treated with probiotics to explore mechanisms by which the gut-brain axis mediates anxiety-like behavior. Our results support the use of probiotics as a promising therapeutic strategy to prevent gut dysbiosis and associated anxiety during opioid withdrawal, with potential implications for improving treatment outcomes in opioid recovery programs.

## Introduction

Opioid use disorder (OUD) is a chronic relapsing condition characterized by compulsive opioid consumption and the development of a negative emotional state upon withdrawal from opioid use.^1^ While individuals report various reasons for initial opioid use, the avoidance of opioid withdrawal is consistently cited as the primary driver for continued use.^2^ Opioid withdrawal syndrome encompasses a range of aversive symptoms caused by the abrupt cessation of chronic opioid use. Physical symptoms of opioid withdrawal syndrome including nausea, diarrhea, fever, and insomnia begin hours after the last dose of opioid and peak after around 72 h.^3^ While physical symptoms usually subside within a week of the last dose, affective symptoms such as anxiety and depression can persist for weeks or months.^4^ Importantly, the severity of these negative emotional states correlates strongly with heightened opioid cravings.^5^ As a result, studies repeatedly associate heightened anxiety during opioid withdrawal with an increased risk of relapse.^6–9^ Despite this, the underlying pathology of emergent anxiety behaviors during opioid withdrawal has not been sufficiently explained. Characterizing the mechanisms underlying these behaviors could facilitate the development of therapeutics aimed at alleviating the negative emotional symptoms of opioid withdrawal, ultimately reducing the risk of relapse.

Previous work from our lab has established the gut microbiome as a driver of several comorbidities associated with opioid use.^10–13^ Chronic opioid use has been shown to induce distinct alterations to the composition of the gut microbiome, leading to a state of dysbiosis that can impact both the immune system and the brain.^13^ Specifically, morphine administration has been linked to reduced alpha diversity in the gut microbiome, indicating decreased microbial richness and evenness, and an increase in potentially pathogenic bacteria such as *Enterococcus faecalis*.^14^ Furthermore, morphine compromises gut epithelial barrier integrity, enhancing the translocation of bacteria and their products, which can trigger pro-inflammatory immune responses.^15^ Gut microbial dysbiosis induced by opioids has been linked to both the development of both morphine analgesic tolerance and somatic withdrawal symptoms. The development of morphine analgesic tolerance is attenuated in germ-free mice – which have no commensal microbiota – or by administration of antibiotics, implicating the gut microbiome as a contributor to this condition.^16^ Studies from our lab and others show that somatic symptoms of morphine withdrawal can be reduced by administration of antibiotics^17^ or by fecal microbiota transplantation from morphine-treated animals,^18^ further establishing gut dysbiosis as a relevant factor in opioid use. Based on this background, we hypothesized that the gut microbiome drives the development of anxiety-like behavior during protracted morphine withdrawal.

Collectively, clinical evidence and animal studies have clearly implicated the gut microbiome as a mediator of anxiety and depression.^19,20^ In humans, irritable bowel syndrome (IBS) is comorbid with anxiety disorders at rates as high as 30–50%.^21^ Analysis of stool samples from patients with anxiety disorders reveals reduced microbial diversity and diminished populations of beneficial bacteria, such as Firmicutes and Tenericutes.^22,23^ Although clinical evidence on the efficacy of probiotics for anxiety and depression is limited,^24^ one study showed that probiotic supplementation alongside methadone maintenance treatment reduced depression severity in OUD patients.^25^ Animal studies using germ-free mice have demonstrated altered anxiety-like behavior compared to conventional mice with an intact gut microbiome, further emphasizing the role of gut health in emotional regulation.^26^ Studies varyingly report either elevated or reduced anxiety-like behavior in germ-free mice,^27,28^ however the connection between the gut microbiome and anxiety is well-established.

Research has begun to explore how gut microbiota influence host physiology not only through their composition, but also through their collective metabolic output.^29^ Microbial metabolites like short-chain fatty acids, bile acids, and tryptophan derivatives can exert widespread effects on the immune system, epithelial barrier, and central nervous system.^30–32^ Fecal microbiota transplantation (FMT), which introduces an entire microbial ecosystem (including metabolites) from a donor to a recipient, is one method for probing causal relationships between microbial communities and host outcomes. Another approach is the use of probiotic therapy, which delivers live microorganisms thought to confer benefit to the host. Several strains included in common probiotic formulations have been shown to alter host immune signaling, reduce systemic inflammation, and influence neurotransmitter pathways relevant to affective behavior.^33,34^ While probiotics do not necessarily reestablish the full complexity of a healthy microbiome, they may influence host physiology through transient activity, microbial interactions, or the production of neuroactive metabolites.^35,36^ In the context of opioid withdrawal, probiotics represent a promising avenue for intervention – not by reversing morphine-induced changes directly, but by modulating the gut ecosystem in a way that buffers against the emergence of withdrawal-associated anxiety.

Our understanding of negative affect associated with protracted opioid withdrawal has been significantly advanced through the use of animal models, particularly mice and rats.^37^ Previous research has effectively modeled opioid withdrawal, demonstrating persistent negative affective behaviors such as anxiety and depression using an array of validated behavioral tests. Heightened anxiety-like and depression-like behavior has been reported in mice following one week of spontaneous morphine withdrawal,^38,39^ lasting four- and seven weeks into protracted opioid withdrawal.^40–42^

The establishment of these mouse models has enabled investigation of the neuropathology that drives the development of anxiety during opioid withdrawal. The amygdala, a brain region critical for emotional regulation, has consistently been implicated in this context.^43–46^ Although the amygdala is highly innervated by serotonin and experimental manipulation of this signaling has been shown to alter anxiety-like behaviors,^47^ this signaling has not yet been implicated in the development of anxiety-like behavior following opioid withdrawal. Considering this background, the amygdala is an attractive target to manipulate therapeutically in effort to prevent the development of anxiety subsequent to opioid withdrawal.

In this study, we provided evidence that protracted morphine withdrawal is associated with gut microbial dysbiosis. Fecal microbiota transplantation from morphine-withdrawn mice to treatment-naive mice was sufficient to induce anxiety-like behavior. Additionally, probiotic therapy during protracted morphine withdrawal prevented anxiety-like behavior. Further, we propose altered serotonin signaling in the amygdala as a potential mechanism of the gut-brain axis that may mediate this behavior. These findings highlight probiotic therapy as a promising, clinically relevant adjunct to mitigate anxiety during opioid detoxification.

## Materials and methods

### Animals

Female and male C57BL/6 mice between the ages of 12–15 weeks were purchased from Taconic Biosciences for use in experiments. Final group sizes for each experiment ranged from *n* = 10–20 per group, depending on the behavioral or molecular endpoint, as detailed in the Results section and figure legends. Total animal numbers and sex distribution were balanced across conditions wherever possible. Mice were housed in a facility which maintains a 12-hour light/dark cycle with constant temperature and humidity with unrestricted access to food and drinking water. All animal procedures were approved by the Institutional Animal Care and Use Committee (IACUC) at the University of Miami and conducted in accordance with the NIH Guide for the Care and Use of Laboratory Animals under approved protocol 23–105.

### Morphine treatment

Mice were subcutaneously implanted with a 75 mg slow-release morphine pellet (or inactive placebo) under isoflurane anesthesia. Briefly, an incision was made along the back of the animal where the pellet was inserted and subsequently closed with surgical staples. Mice were removed from anesthesia and returned to home cage. After 72 h, surgical staples were removed and the same incision was reopened to facilitate removal of the pellet, inducing spontaneous withdrawal. As before, the incision was closed with surgical staples and mice were removed from anesthesia and returned to their home cage.

### Fecal microbiota transplantation

Before fecal microbiota transplantation (FMT), FMT recipient mice were first pre-treated with antibiotics to deplete their gut microbiome. FMT recipients received twice daily oral gavage of an antibiotic cocktail (neomycin: 100 mg/kg, metronidazole: 100 mg/kg, vancomycin: 50 mg/kg) for 7 d. Additionally, home-cage drinking water was supplemented with 1 mg/ml ampicillin. FMT donor mice were subjected to protracted morphine withdrawal, or placebo treatment, as described above. On the eighth day following withdrawal, colon contents were harvested and processed for transplantation later the same day. Fresh colon contents from FMT donor mice were diluted in sterile PBS at a ratio of 1 ml PBS/150 mg and vortexed for one minute to homogenize. The mixture was filtered through a 75 μm cell strainer to separate out large solids. Approximately 36 h after the last dose of antibiotic cocktail, FMT recipient mice received 150 μl of the FMT solution by oral gavage. FMT recipient mice received a second dose of FMT six h after the first dose.

### Probiotic treatment

VSL#3 double strength powder (900 billion CFU per packet) was purchased from VSL Probiotics for use in probiotic treatments. The VSL#3 probiotic blend contains the following bacterial strains: *Streptococcus thermophilus, Bifidobacterium breve, Bifidobacterium lactis, Lactobacillus acidophilus, Lactobacillus plantarum, Lactobacillus paracasei, Lactobacillus helveticus*. VSL#3 powder was suspended in nuclease free water at a ratio of 50 mg VSL#3 per 150 µL water, yielding a concentration of approximately 1.6 × 10^9^ CFU per 150 µL dose, which was administered by oral gavage. Probiotic therapy was administered once daily at 5pm during protracted withdrawal, initiated the day of pellet removal and continued until the sixth day following pellet removal, for a total of six treatments.

### Behavioral testing

On the sixth day following pellet removal, mice completed the open field test as an assessment of anxiety-like behavior. Mice were moved from the housing facility to the testing room at 6pm and allowed to acclimate to the testing environment for two h before testing. The testing environment was kept under dark-light conditions with a white noise machine to cover background noise. Mice were tested using the Photobeam Activity System (PAS)-Open Field Test from San Diego Instruments, which employs a 16 × 16photobeam floor grid to track activity. Mice were placed in the center of the open field and allowed to roam freely for 30 minutes. Activity was recorded by the number of beam breaks, and activity in the center was defined by beam breaks in the center 4 × 4 of the grid. After the completion of testing, mice were returned to the housing facility.

On the seventh day following pellet removal, mice completed the elevated plus maze as a further assessment of anxiety-like behavior. Mice were moved to the testing environment at 6pm and allowed to acclimate to the testing environment for two h before testing. The testing environment was kept under red-light conditions with a white noise machine to cover background noise. Mice were placed in the center of the maze, facing an open arm, and allowed to explore the maze for 5 minutes. Activity was recorded by a camera positioned overhead which recorded video of the test. Videos were subject-blinded and manually scored. Time spent in the open and closed arms of the maze, as well as the number of entrances into the open and closed arms were recorded.

### Collection of biological specimens

The morning following behavioral testing, mice were sacrificed for collection of biological specimens. Mice were euthanized first by carbon dioxide chamber, followed by cervical dislocation. Amygdala tissue was collected following a modified protocol based on previously published methods.^48,49^ Briefly, brains were sectioned into 1 mm-thick coronal slices. The amygdala was isolated from the slice corresponding approximately to bregma −1.0 mm to −2.0 mm. Dissections were guided by visual landmarks, including the appearance of the hippocampus and external capsule, as described in Jia et al., to ensure consistency across samples.^48^ Intestinal contents were harvested from the colon. Samples were flash frozen on dry ice and stored at −80°C until analysis.

### DNA extraction and 16S rRNA gene sequencing

Intestinal contents were flash frozen on dry ice after collection and were stored at −80°C until ready for analysis. DNA was extracted from fecal samples using the DNeasy PowerSoil Pro kit (Qiagen; catalog no. 47016). Two extraction controls were included in sequencing to prevent contamination from the kit reagents. Sequencing was conducted by the University of Minnesota Genomics Center. The V4 region of the 16S rRNA gene was amplified by PCR using the forward primer 515F (GTGCCAFCMGCCGCGGTAA) and the reverse primer 806 R (GGACTACHVGGGTWTCTAAT), along with Illumina adaptors and molecular barcodes, resulting in 427-bp amplicons. These amplicons were sequenced on the Illumina MiSeq version 3 platform, producing 300-bp paired-end reads.

### Microbiome analysis

Demultiplexed sequence reads were clustered into amplicon sequence variants (ASVs) with the DADA2 package (version 1.32.0)^50^ in R (version 4.4.0) and RStudio (Build 764). The steps of the DADA2 pipeline include error filtering, trimming, learning of error rates, denoising, merging of paired reads, and removal of chimeras. During trimming, the forward and reverse reads were truncated at positions 220 and 190 to remove low-quality tails using filterAndTrim function, with filtering parameters: truncLen=c(220,190), trimLeft=c(19,20), maxN = 0, maxEE=c(2,2), truncQ = 2, rm.phix=TRUE. ASVs were assigned taxonomically at the species level using a naive Bayesian classifier^51^ in DADA2 with the SILVA reference database (release 138.1).^52^ The ASV and taxonomy tables were imported in MicrobiomeAnalyst^53^ to create alpha and beta diversity plots, taxonomy bar plots, and linear discriminant analysis effect size (LEfSe)^54^ plots. Low count and low variance ASVs were filtered with the default threshold. Total sum scaling was used for normalization. The threshold on the logarithmic LDA score for discriminative features was set to 2. The cutoff for *p*-value was set to 0.05 for LEfSe analysis. The Mann-Whitney test was used to detect if alpha diversity differed across treatments. Permutational multivariate analysis of variance (PERMANOVA) was used to detect if beta diversity differed across treatments.

### Metabolomic analysis

Collected colon contents were flash frozen and stored at −80°C until being submitted to Creative Proteomics for untargeted metabolomics analysis. Sample preparation, metabolite extraction, and UPLC-MS analysis were performed by Creative Proteomics following standardized protocols. Briefly, samples were extracted using 80% methanol, followed by bead homogenization, sonication, precipitation, and centrifugation. Supernatants were spiked with internal standards and filtered for LC-MS. Chromatographic separation was performed using a Vanquish Flex UPLC system coupled with a Q Exactive Plus Orbitrap mass spectrometer (Thermo Fisher Scientific) using both positive and negative electrospray ionization (ESI+ and ESI–) modes. The LC-MS gradient, column conditions, and mass spectrometry parameters followed the manufacturer’s established protocol (see Creative Proteomics Report CPMS11072402–01). Raw metabolite intensities were normalized by dividing each metabolite’s peak area by the total ion current (TIC) for each sample and multiplying by 1 million, as specified by the provider. Principal component analysis (PCA) and volcano plots were generated in R using the ggplot2 and stats packages to visualize global group separation and identify differentially abundant metabolites. Metabolite enrichment analysis was performed using the Quantitative Enrichment Analysis (QEA) module in MetaboAnalyst 6.0,^55^ using the KEGG pathway library for Mus musculus. Relative abundance of metabolites between groups were compared using the Mann-Whitney test.

### Transcriptome analysis

Collected samples of amygdala tissue were submitted for bulk RNA sequencing. RNA sequencing was performed by Novogene Co. Raw data (raw reads) of fastq format were first processed through Novogene in-house perl scripts. In this step, clean data (clean reads) were obtained by removing reads containing adapter, reads containing poly-N and low-quality reads from raw data. At the same time, Q20, Q30 and GC content of the clean data were calculated. All the downstream analyses were based on clean data with high quality. Index of the reference genome was built using Hisat2 (v2.0.5)^56^ and paired-end clean reads were aligned to the reference genome using Hisat2. FeatureCounts (v1.5.0)^57^ from Subread package was used to count the reads numbers mapped to each gene. FPKM of each gene was calculated based on the length of the gene and reads count mapped to this gene. Differential expression analysis was performed using the DESeq2^58^ package (1.20.0). The resulting *p*-values were adjusted using the Benjamini and Hochberg approach for controlling false discovery rate. Genes with an adjusted *p*-value ≤0.05 found by DESeq2 were assigned as differentially expressed. Differentially expressed genes were subsequently analyzed using QIAGEN Ingenuity Pathway Analysis (IPA) for canonical pathways enrichment analysis. Fisher’s exact test was utilized in all those analyses to identify the signaling and metabolic pathways significantly associated with differentially expressed genes. Pathways with a *p*-value <0.05 and a |z-score| >2 are considered significant.

### RT^2^ Profiler array

Total RNA was extracted from mouse amygdala tissue using the RNeasy® Universal Plus Mini Kit (QIAGEN), according to the manufacturer’s instructions. RNA quality and quantity were assessed using a NanoDrop spectrophotometer, and only high-quality RNA (A260/A280 ~ 2.0) was used for downstream applications. cDNA was synthesized from RNA using the RT^2^ First Strand Kit (QIAGEN).

Gene expression profiling was performed using the RT^2^ Profiler Mouse Dopamine & Serotonin Pathway PCR Array (GeneGlobe ID: PAMM-158ZF). The cDNA was mixed with RT^2^ SYBR® Green Mastermix and added to the 96-well array plate containing pathway-focused gene-specific primers. Real-time PCR was conducted using the Roche LightCycler® 480 thermocycler. Cycling conditions followed the manufacturer’s recommended protocol (Table 5 from the RT^2^ Profiler PCR Array Handbook): 95°C for 10 min, followed by 45 cycles of 95°C for 15 s and 60°C for 1 min. Threshold cycle (Ct) values were calculated using the LightCycler software, and gene expression changes were analyzed using the ΔΔCt method. Data were normalized to the average Ct values of five housekeeping genes included on the array. Fold-change values were calculated using the RT^2^ Profiler PCR Array Data Analysis Excel template provided by QIAGEN.

### Statistical analysis

GraphPad Prism version 10.3.1 (GraphPad Software, Boston, Massachusetts USA, www.graphpad.com) was used for statistical analysis of behavioral data, as well as correlational- and linear regression modeling of transcriptome data. Details for statistical tests conducted for each experiment are found in figure legends. Outliers were identified using the ROUT method, with Q set to 1%.

## Results

### Protracted withdrawal from chronic morphine treatment is associated with heightened anxiety-like behavior in female and male mice

**Figure 1.**
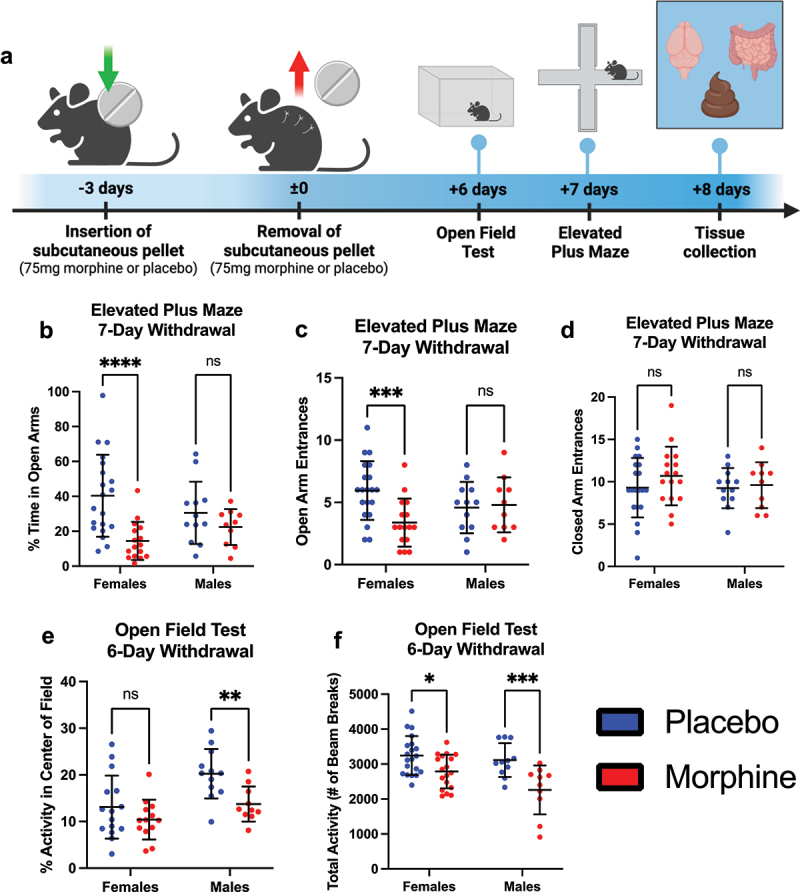
Protracted morphine withdrawal is associated with elevated anxiety-like behavior, with sex-differences. (a) general experimental paradigm for morphine/placebo withdrawal and behavioral testing. (b) percent time spent in the open arms of the elevated plus maze for female and male mice. (c) number of entrances into the open arms of the elevated plus maze for female and male mice. (d) number of entrances into the closed arms of the elevated plus maze for female and male mice. (e) percent activity in the center of the open field test for female and male mice. (f) total activity measured by number of beam breaks in the open field test for female and male mice. Symbols represent individual mice; line and error bars represent mean and standard deviation; n_Female_ = 20 placebo, 16 morphine; n_Male_ = 12 placebo, 10 morphine;. ns = nonsignificant, *p < 0.05, **p < 0.01, ***p < 0.001, ****p < 0.0001 using two-way ANOVA followed by Fisher’s least significant difference post hoc test.

To model protracted morphine withdrawal, female (*n* = 20 per group) and male (*n* = 15 per group) mice were implanted with a 75 mg slow-release subcutaneous morphine or placebo pellet. Pellets were removed after 72 hours to initiate spontaneous withdrawal. On days six and seven post-removal, mice underwent the elevated plus maze (EPM) and open field test (OFT) to assess anxiety-like behavior (Figure 1(a)). Four morphine-treated females and five males died prior to pellet removal. One placebo-treated male and two cage-mates were euthanized due to aggression-related injuries.

The EPM served as the primary behavioral assay. Two-way ANOVA revealed a significant main effect of treatment on time spent in the open arms (F(1,54) = 12.7, *p* = 0.0008), with no effect of sex (F(1,54) = 0.0371, *p* = 0.848) and a trend toward a treatment × sex interaction (F(1,54) = 3.48, *p* = 0.0675). Post hoc tests showed that morphine-withdrawn females spent significantly less time in the open arms compared to placebo-treated females (*t* = 4.40, *p* < 0.0001), whereas males showed no significant difference (*t* = 1.08, *p* = 0.491) (Figure 1(b)).

For number of open arm entries, ANOVA again revealed a significant effect of treatment (F(1,54) = 4.04, *p* = 0.0494) and a significant treatment × sex interaction (F(1,54) = 5.66, *p* = 0.0209), with no main effect of sex (F(1,54) = 0.00247, *p* = 0.961). Post hoc analysis confirmed that morphine-withdrawn females made significantly fewer entries into the open arms than placebo-treated females (*t* = 3.56, *p* = 0.0016), while males again showed no difference (*t* = 0.235, *p* = 0.815) (Figure 1(c)). Closed arm entries did not differ by treatment in either sex (females: *t* = 1.31, *p* = 0.197; males: *t* = 0.258, *p* = 0.797) (Figure 1(d)), suggesting no general motor deficits in the EPM.

In the OFT, ANOVA revealed a significant effect of treatment (F(1,46) = 9.18, *p* = 0.004) and sex (F(1,46) = 11.9, *p* = 0.0012) on time spent in the center, but no interaction (F(1,46) = 1.58, *p* = 0.215). Morphine-withdrawn males spent significantly less time in the center than placebo-treated males (*t* = 2.86, *p* = 0.0063), whereas females showed no significant difference (*t* = 1.34, *p* = 0.187) (Figure 1E).

For total activity, there were main effects of treatment (F(1,55) = 19.1, *p* < 0.0001) and sex (F(1,55) = 4.83, *p* = 0.0322), with no interaction (F(1,55) = 1.76, *p* = 0.190). Both morphine-withdrawn females (*t* = 2.55, *p* = 0.0134) and males (*t* = 3.55, *p* = 0.0008) showed significantly reduced overall locomotion compared to placebo controls (Figure 1(e)).

Together, these data suggest that morphine withdrawal induces sex-specific anxiety-like behaviors, with female mice exhibiting classic anxiety patterns in the EPM, and male mice showing anxiety-like reductions in center activity in the open field. Both sexes displayed reduced general activity following withdrawal.

### Protracted withdrawal from chronic morphine treatment results in gut microbial dysbiosis in female and male mice

**Figure 2.**
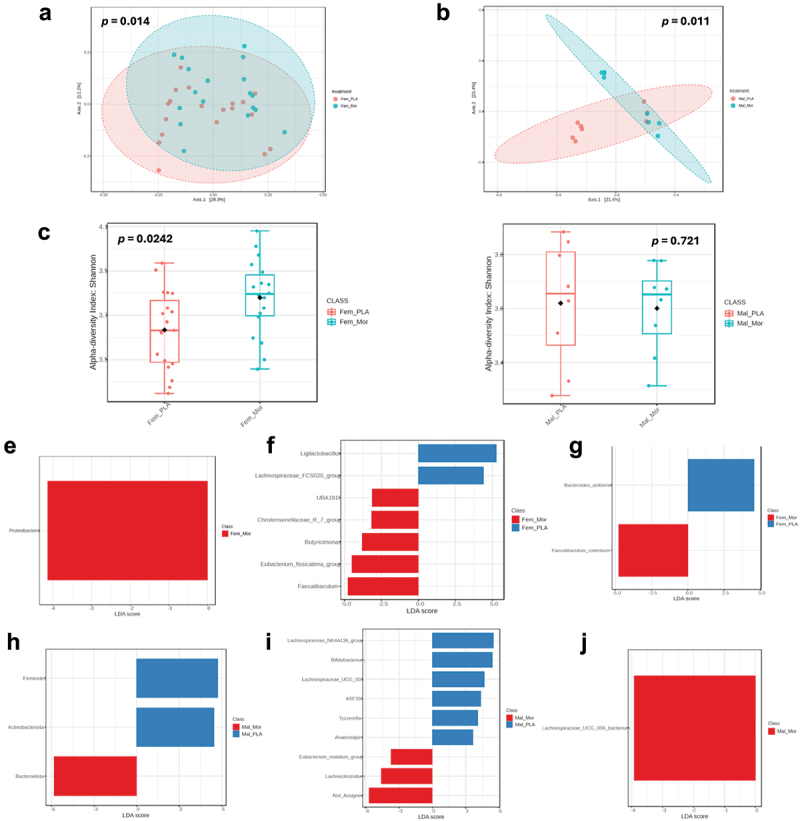
Gut microbial dysbiosis associated with protracted morphine withdrawal in female and male mice. (a) Principal coordinate analysis plot of Bray-Curtis distance (measure of β-diversity) for female mice withdrawn from morphine and placebo controls. (b) Principal coordinate analysis plot of Bray-Curtis distance (measure of β-diversity) for male mice withdrawn from morphine and placebo controls. (c) α-diversity plotted by Shannon index for female mice withdrawn from morphine and placebo controls. (d) α-diversity plotted by Shannon index for male mice withdrawn from morphine and placebo controls. (e) LEfSe plot for phyla enriched in morphine withdrawn-female mice. (f) LEfSe plot for genera enriched and depleted in morphine-withdrawn female mice. (g) LEfSe plot for species enriched and depleted in morphine-withdrawn female mice. (h) LEfSe plot for phyla enriched and depleted in morphine-withdrawn male mice. (i) LEfSe plot for genera enriched and depleted in morphine-withdrawn male mice. (j) LEfSe plot for species enriched in morphine-withdrawn male mice. Fem_PLA = female placebo controls (*n*=19), Fem_Mor = female morphine withdrawal (*n*=18), Mal_PLA = male placebo controls (*n*=8), Mal_Mor = male morphine withdrawal (*n*=8).

Previous studies from our lab have demonstrated gut dysbiosis associated with morphine dependence,^14^ and a partial recovery over the first 24 h of morphine withdrawal,^17^ however the state of the gut microbiome in the protracted stage of morphine withdrawal has not been described. To bridge this knowledge gap, we utilized 16S rRNA sequencing of colon contents collected from mice following behavioral testing. 16S rRNA sequencing demonstrated lasting alterations to the composition of the gut microbiome, consistent with gut microbial dysbiosis, in both female and male mice during protracted withdrawal from chronic morphine treatment. Beta diversity analysis, reported here by Bray-Curtis dissimilarity, revealed a significant shift in the composition of the gut microbiome following protracted morphine withdrawal in both female (F = 2.61, *p* = 0.014) (Figure 2(a)) and male mice (F = 30.1, *p* = 0.011) (Figure 2(b)). More specifically, alpha diversity plotted by the Shannon index was increased in female mice withdrawn from morphine compared to placebo controls (U = 97, *p* = 0.0242) (Figure 2(c)), indicating an increase in species richness and evenness following protracted morphine withdrawal. There was no such alteration in alpha diversity associated with morphine withdrawal in male mice (U = 36, *p* = 0.721) (Figure 2(d)). This increase in alpha diversity seen in female mice withdrawn from morphine stands in contrast to the decrease in alpha diversity associated with morphine dependence,^14^ and represents an expansion during protracted withdrawal that overtakes placebo control levels.

Female mice withdrawn from morphine exhibited an enrichment of the phylum Proteobacteria compared to placebo controls (Figure 2(e)). At the genus level, female mice withdrawn from morphine showed an expansion of *Faecalibaculum*, *Eubacterium fissicatena*, *Butyricimonas*, *Christensenellaceae R7*, and *UBA 1819* in addition to a depletion of *Ligilactobacillus* and *Lachnospiraceae FCS020* (Figure 2(f)). At the species level, *Faecalibaculum rodentium* was expanded and *Bacteroides uniformis* was depleted in female mice withdrawn from morphine (Figure 2(g)).

Male mice withdrawn from morphine exhibited an enrichment of the phylum Bacteroidota, and depletion of the phyla Actinobacteria and Firmicutes compared to placebo controls (Figure 2(h)). At the genus level, male mice withdrawn from morphine showed an expansion of *Lachnoclostridium*, *Eubacterium nodatum*, and an unassigned bacterial genus while the genera *Anaerostipes*, *Tyzzerella*, *ASF356*, *Lachnospiraceae UCG 001*, *Bifidobacterium*, and *Lachnospiraceae NK4A136* were depleted (Figure 2(i)). The species *Lachnospiraceae UCG 006* bacterium was expanded in male mice withdrawn from morphine (Figure 2(j)).

Expansion of Proteobacteria coupled with the depletion of probiotic groups such as *Bacteroides uniformis* provided evidence of gut dysbiosis in female mice withdrawn from morphine. The altered ratio of Bacteroidota and Firmicutes abundance observed in male mice withdrawn from morphine, as well as depletion of probiotic flora such as *Bifidobacterium*, similarly indicated a dysbiotic gut microbiome.

### Metabolomic analysis of colon contents reveals altered tryptophan metabolism associated with protracted morphine withdrawal

**Figure 3.**
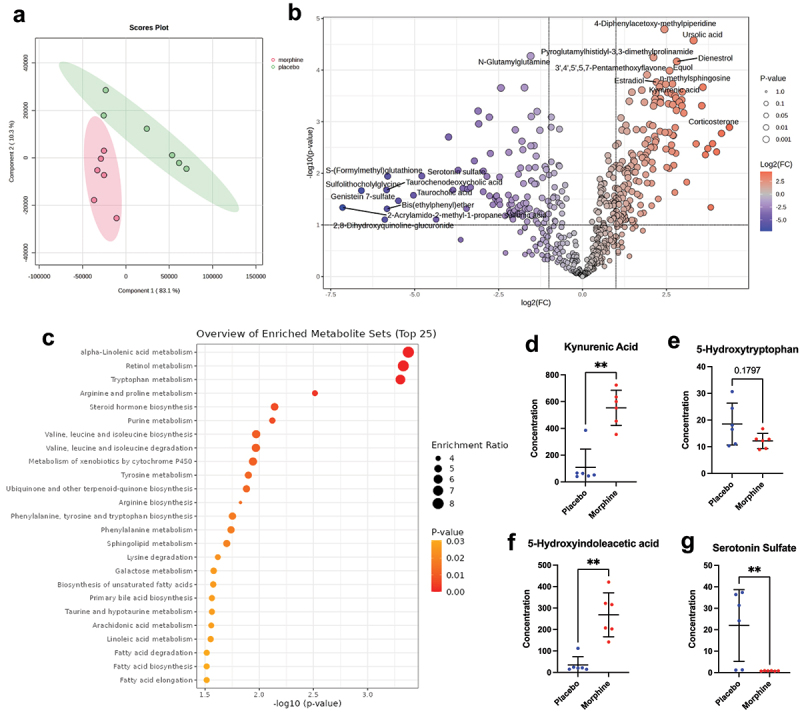
Morphine withdrawal is associated with a distinct metabolomic profile characterized by alterations in tryptophan metabolism. (a) partial least squares discriminant analysis (PLS-DA) of colon metabolite profiles from morphine-withdrawn mice (morphine, red) and placebo controls (placebo, green). (b) volcano plot displaying differentially abundant metabolites in morphine withdrawal compared to placebo. (c) pathway enrichment analysis of differentially abundant metabolites between morphine withdrawal and placebo groups, highlighting significantly altered biochemical pathways. (d-g) relative abundance of key tryptophan pathway metabolites in colon contents from morphine withdrawn mice (morphine) and placebo controls (placebo), including kynurenic acid, 5-hydroxytryptophan, 5-hydroxyindoleacetic acid, and serotonin sulfate. *n*=6/group; **p<0.01 using the Mann-Whitney test.

Metabolites produced by the gut microbiome play a crucial role in gut-brain communication, influencing neurotransmission, immune function, and behavior.^32^ Therefore, to identify metabolic mechanisms linking gut dysbiosis to anxiety-like behavior in morphine withdrawal, we submitted colon contents from female mice withdrawn from either morphine (*n* = 6) or placebo (*n* = 6) for untargeted metabolomic analysis. Partial Least Squares Discriminant Analysis (PLS-DA) was performed to visualize differences in the metabolomic profiles of morphine-withdrawn (morphine) and placebo-withdrawn (placebo) mice. The resulting plot (Figure 3(a)) shows clear separation between the groups, indicating that morphine withdrawal induces a distinct metabolic signature. A total of 585 metabolites were differentially abundant between morphine-withdrawn and placebo-withdrawn mice, with 345 metabolites elevated in the morphine group and 240 elevated in the placebo group (Figure 3(b)).

To identify broader metabolic pathways affected by morphine withdrawal, we performed pathway enrichment analysis which revealed significant alterations in several key biochemical pathways, including retinol metabolism, tryptophan metabolism, and steroid hormone biosynthesis (Figure 3(c)).

Among the most significantly enriched metabolic pathways in morphine withdrawal, tryptophan metabolism emerged as a potentially relevant mechanism underlying the development of anxiety-like behavior, given its role in regulating central serotonin availability.^59^ To contextualize the impact of morphine withdrawal on tryptophan metabolism, we examined the relative abundance of key metabolites within this pathway (Figures 3(d-g)). While the difference in 5-hydroxytryptophan (5-HTP) levels between groups was not statistically significant, a downward trend in the morphine withdrawal group suggests a possible reduction in the availability of this serotonin precursor. In contrast, the levels of kynurenic acid – a key metabolite in the kynurenine pathway – were significantly elevated in morphine-withdrawn mice, indicating increased tryptophan catabolism via this non-serotonergic route. This metabolic shift was further supported by a significant increase in 5-hydroxyindoleacetic acid (5-HIAA), the primary serotonin degradation product, in morphine-withdrawn mice. Additionally, serotonin sulfate, a conjugated form of serotonin that may serve as a storage or detoxification mechanism, was dramatically reduced in morphine withdrawal, suggesting an overall decrease in serotonin stabilization. Together, these findings support the hypothesis that in the context of morphine withdrawal, tryptophan is preferentially diverted toward the kynurenine pathway, potentially limiting its availability for serotonin synthesis and contributing to withdrawal-associated

anxiety-like behavior

### Fecal microbiota transplantation from morphine-withdrawn mice to treatment-naive mice induces anxiety-like behavior

**Figure 4.**
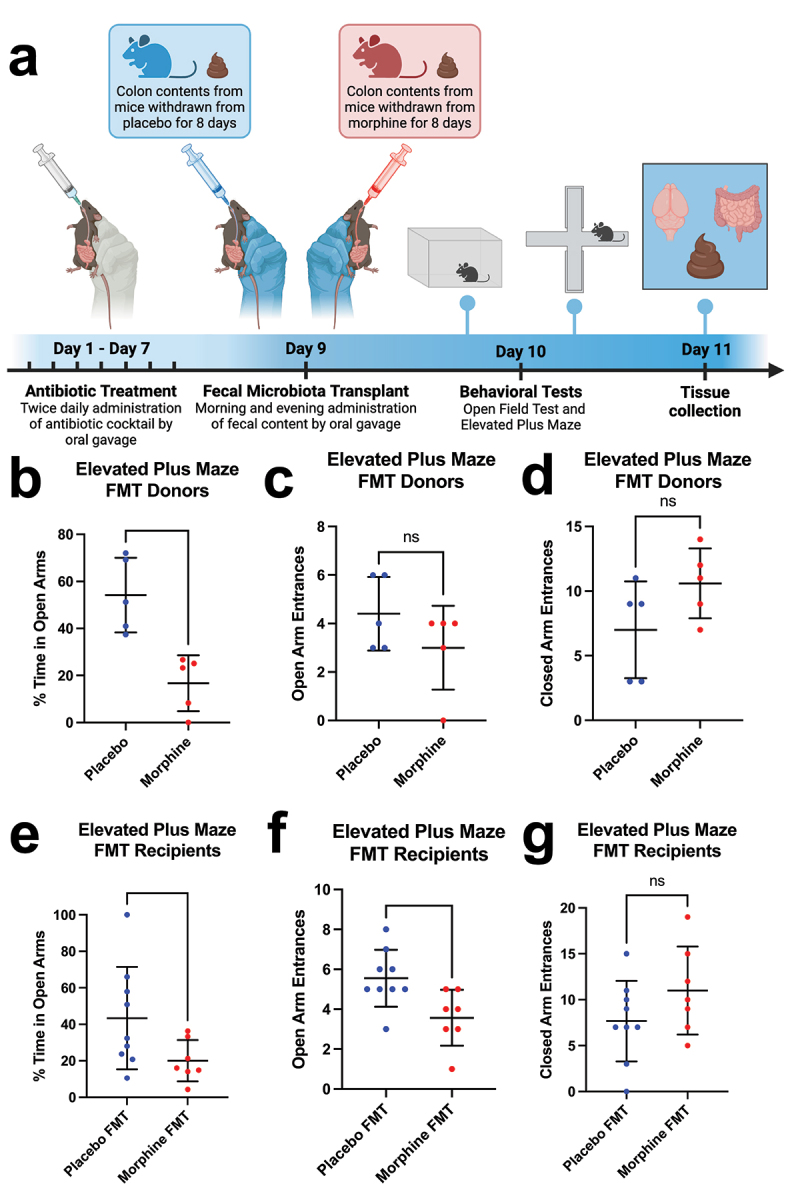
Fecal microbiota transplantation (FMT) from mice withdrawn from morphine results in increased anxiety-like behavior in the elevated plus maze. (a) experimental paradigm for FMT and behavioral testing. (b) percent time spent in the open arms of the elevated plus maze for FMT donor mice withdrawn from morphine and placebo controls. (c) number of entrances into the open arms of the elevated plus maze for FMT donor mice withdrawn from morphine and placebo controls. (d) number of entrances into the closed arms of the elevated plus maze for FMT donor mice withdrawn from morphine and placebo controls. (e) percent time spent in the open arms of the elevated plus maze for mice who received FMT from morphine withdrawn mice and placebo controls. (f) number of entrances into the open arms of the elevated plus maze for mice who received FMT from morphine withdrawn mice and placebo controls. (g) number of entrances into the closed arms of the elevated plus maze for mice who received FMT from morphine withdrawn mice and placebo controls. n_Donors_= 5 placebo, 5 morphine; n_Recipients_= 9 placebo, 7 morphine; symbols represent individual mice; line and error bars represent mean and standard deviation; ns= nonsignificant, *p<0.05, **p<0.01 using unpaired t-test with Welch’s correction.

After observing gut dysbiosis, altered gut-derived metabolites, and anxiety-like behavior following protracted morphine withdrawal, we set out to interrogate a causal relationship between these adverse outcomes. Specifically, we sought to determine whether the dysbiotic microbial and metabolic environment associated with morphine withdrawal was sufficient to induce anxiety-like

behavior in morphine-naive mice. To test this, we devised an experimental paradigm utilizing fecal microbiota transplantation (FMT) from female mice withdrawn from morphine or placebo controls into treatment-naive female recipients (Figure 4(a)). FMT donor mice completed behavioral testing during protracted morphine withdrawal (*n* = 10), or placebo treatment (*n* = 10), as previously described. Five mice from each group exhibiting the most significantly different behavior on the elevated plus maze (Figures 4(b-d)) were selected to have their gut microbiome transplanted to antibiotic-treated FMT recipients (*n* = 10/group). One mouse in the Placebo FMT group and three mice in the Morphine FMT group made fewer than five combined entrances into either the open or closed arms of the elevated plus maze, and thus were excluded as outliers.

Mice that received FMT from donors withdrawn from morphine (Morphine FMT) spent significantly less time in the open arms of the elevated plus maze than mice that received FMT from donors treated with placebo (Placebo FMT) (*t* = 2.264, *p* = 0.0447) (Figure 4(d)). Additionally, Morphine FMT mice made fewer entrances into the open arms of the maze than Placebo FMT mice (*t* = 2.787, *p* = 0.0145) (Figure 4(f)). There were no significant behavioral differences found in the open field test (Supplemental Figure 1). These results indicate that Morphine FMT mice show elevated anxiety-like behavior relative to the Placebo FMT group, suggesting that the effect of the gut microbiome and gut-derived metabolites associated with morphine withdrawal alone is sufficient to induce anxiety-like behavior.

### Administration of probiotics during morphine withdrawal rescues the induction of anxiety-like behavior in female mice

**Figure 5.**
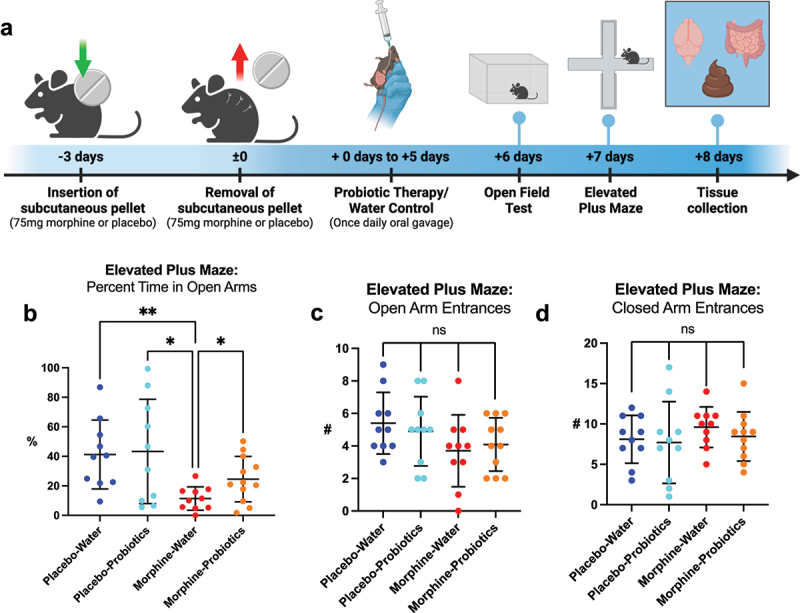
Probiotic therapy during protracted withdrawal partially rescues the development of anxiety-like behavior during protracted morphine withdrawal. (a) experimental paradigm for probiotic therapy during protracted morphine withdrawal and behavioral testing. (b) percent time spent in the open arms of the elevated plus maze. (c) number of entrances into the open arms of the elevated plus maze. (d) number of entrances into the closed arms of the elevated plus maze. Symbols represent individual mice; line and error bars represent mean and standard deviation. n_Placebo-Water_ = 10, n_Placebo-Probiotics_ = 10, n_Morphine-Water_ = 10, n_Morphine-Probiotics_ = 12; *p < 0.05, **p < 0.01 using unpaired t-test with Welch’s correction.

After establishing gut dysbiosis associated with protracted morphine withdrawal, we next investigated the gut microbiome as a therapeutic target to prevent the development of anxiety-like behavior subsequent to opioid withdrawal. Because morphine withdrawal impacted female mice more than male mice in terms of anxiety-like behavior and gut dysbiosis in the above experiments, it was decided to utilize female mice for the following experimental design. Based on the results of 16S sequencing of the gut microbiome, we decided to apply a probiotic therapy to counteract the expansion of dysbiotic flora and depletion of probiotic flora observed in female mice withdrawn from morphine treatment. To this end, female mice (n = 10–15 per group) underwent spontaneous withdrawal as previously described, with the addition of once-daily administration of VSL#3 probiotic blend (or water control) by oral gavage throughout the protracted withdrawal phase until behavioral testing (Figure 5(a)). Four mice assigned to the morphine-water group and three mice assigned to the morphine-probiotics group perished before pellet removal. One mouse assigned to the morphine-water group removed its subcutaneous morphine pellet before 72 hours and was not included in analysis.

Comparing time spent in the open arms of the elevated plus maze, Welch’s ANOVA test determined a significant difference between the means of the four treatment groups (F(3,18.8) = 7.53, *p=*0.0017). According to *post hoc* analysis, the morphine-water group spent significantly less time in the open arms of the maze than the placebo-water group (*t* = 3.83, *p* = 0.0028) (Figure 5(b)), confirming the induction of anxiety-like behavior in the positive control group. Importantly, the morphine-withdrawn females that received probiotic therapy spent significantly more time in the open arms of the maze than the morphine-water group (*t* = 2.59, *p* = 0.0191) (Figure 5(b)). This indicates lower anxiety-like behavior in morphine treated females following probiotic therapy. Of note, there was no significant difference in time spent in the open arms of the maze between the morphine-probiotics group and the placebo-probiotics group (*t* = 1.56, *p* = 0.146) (Figure 5(b)). One-way ANOVA analysis revealed no significant difference among the mean number of entrances into the open arms of the maze between any of the four treatment groups (F(3,37) = 1.53, *p* = 0.222) (Figure 5(c)). Additionally, one-way ANOVA showed no significant difference in entrances into the closed arms of the maze among the four groups (F(3,37) = 0.540, *p* = 0.658) (Figure 5(d)). These results show a rescue of the induction of anxiety-like behavior associated with protracted morphine withdrawal in female mice treated with VSL#3 probiotic blend.

In the open field test, there were no significant differences in levels of activity in the center of the field or total activity levels between any of the four treatment groups [Supplementary Figure S2A, S2B]. This was expected based on previous experiments using female mice.

Results from the elevated plus maze, our primary assessment of anxiety-like behavior, indicate that probiotic therapy during protracted withdrawal from chronic morphine treatment rescues the induction of anxiety-like behavior, implicating the gut microbiome as a driver of this behavior.

### Protracted morphine withdrawal induces transcriptomic changes in the amygdala of female and male mice

**Figure 6.**
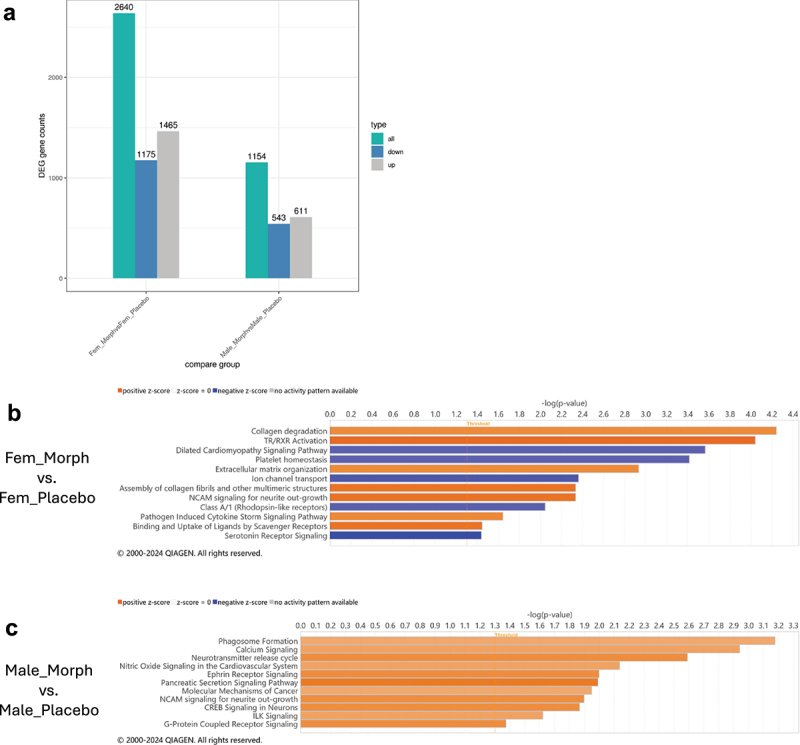
Transcriptional alterations to the amygdala associated with protracted morphine withdrawal in female and male mice. (a) differentially expressed gene (DEG) counts for Fem_Morph vs. Fem_Placebo and Male_Morph vs. Male_Placebo. (b) pathways identified by IPA as upregulated and downregulated in Fem_Morph vs. Fem_Placebo. (c) pathways identified by IPA as upregulated in Male_Morph vs. Male_Placebo. Fem_Morph = female morphine withdrawal (*n*=6), Fem_Placebo = female placebo controls (*n*=6), Male_Morph = male morphine withdrawal (*n*=6), Male_Placebo = male placebo controls (n*=*6).

The gut microbiome can have significant impacts on the brain and behavior, and the amygdala has been identified as a critical signaling node across the gut-brain axis.^60^ We endeavored to investigate potential mechanisms that mediate the impact of morphine withdrawal-induced gut dysbiosis on anxiety-like behavior by assessing the transcriptomic state of the amygdala during protracted withdrawal. To assess transcriptional alterations in the brain associated with protracted morphine withdrawal, amygdala samples collected from female and male mice (*n* = 6 per group) in the above paradigm of withdrawal and behavioral testing (Figure 1(a)) were submitted for bulk RNA-sequencing. Analysis of differential expressed genes (DEGs) between morphine-withdrawn and placebo-treated mice reveals a greater degree of transcriptional alteration in female mice than male mice (Figure 6(a)). 2640 genes were identified as differentially expressed in female mice withdrawn from morphine compared to placebo controls, whereas 1154 DEGs were identified in morphine-withdrawn male mice compared to placebo controls. Overall, the pattern of differential expression favored a greater degree of upregulated- than downregulated genes for both female and male mice. Interestingly, this pattern resembles the results from behavioral testing which indicate more significant anxiety-like behavior in female mice withdrawn from morphine compared to male mice.

In female mice withdrawn from chronic morphine treatment, a number of pathways were identified by Ingenuity Pathway Analysis (IPA) as either significantly activated or inhibited (Figure 6(b)). Of particular interest was the inhibition of serotonin receptor signaling, due to the well-established connection between serotonin and anxiety.^61^ On this signaling pathway, genes downregulated in females withdrawn from morphine withdrawal included genes encoding multiple serotonin receptors, such as *Htr2c* and *Htr5b*, as well as the adrenergic receptor *Adra2a*. Interestingly, despite the more significant pattern of gene downregulation on the serotonin receptor signaling pathway, upregulation of several genes associated with this pathway was observed. These upregulated genes included *Htr6* and *Htr1d* which also encode for serotonin receptors, along with the adrenoreceptor *Adra2b*. The identification of these pathways provides insight into potential mechanisms that regulate the development of anxiety-like behavior subsequent to morphine withdrawal.

Among male mice withdrawn from morphine, IPA showed only significant activated pathways with no significantly inhibited signaling pathways (Figure 6(c)). Included in these signaling pathways was activation of G-protein coupled receptor signaling. On this pathway, upregulation of the genes encoding for adrenoceptor alpha 1B (*Adra1b*) as well as dopamine receptor D4 (*Drd4*) was observed, as well as downregulation of *Htr7* which encodes for a serotonin receptor.

### Probiotic treatment during protracted morphine withdrawal is associated with transcriptomic changes in the amygdala of female mice

**Figure 7.**
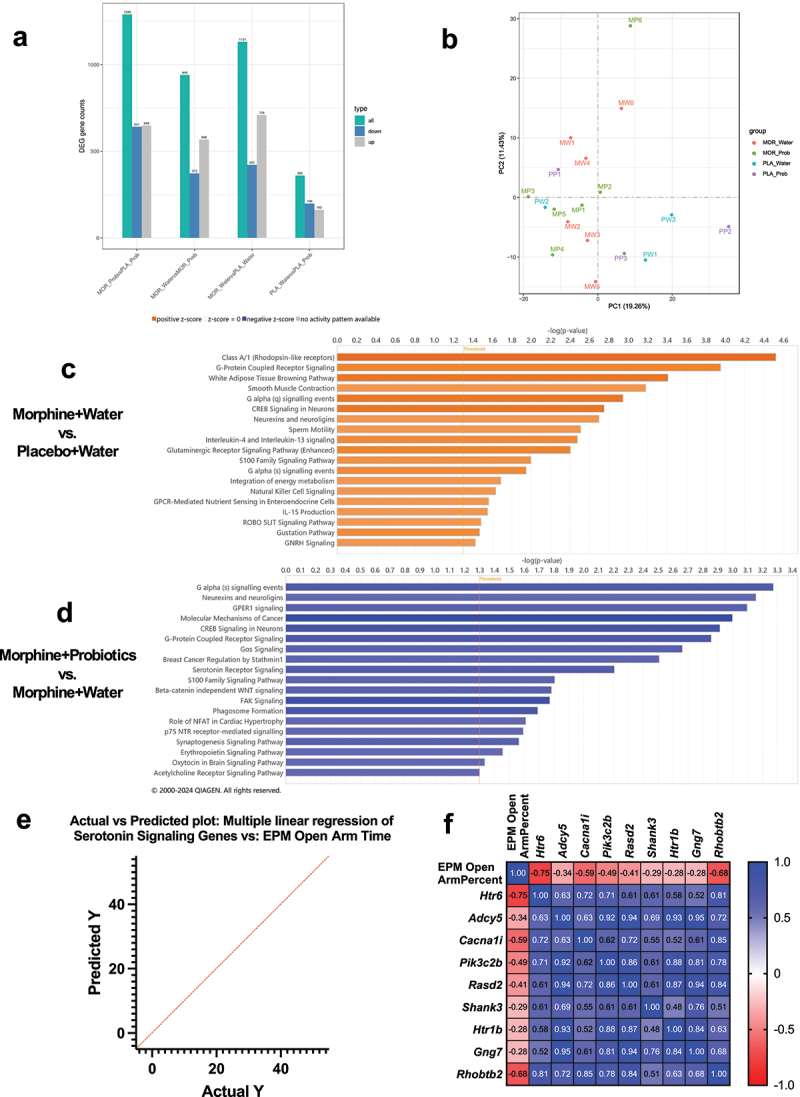
Transcriptional alterations in the amygdala during protracted morphine withdrawal and probiotic treatment. (a) differentially expressed gene (DEG) counts across all experimental comparisons. (b) Principal component analysis (PCA) of amygdala transcriptomes across all groups reveals clustering by treatment condition. (c) top differentially expressed pathways in morphine withdrawal identified using Ingenuity pathway analysis (IPA). (d) top differentially expressed pathways following probiotic treatment during withdrawal. (e) multiple linear regression model showing the relationship between altered serotonin signaling genes and percent time spent in the open arms of the elevated plus maze (EPM). (f) correlation matrix showing the relationship between serotonin signaling genes downregulated by probiotic treatment and EPM open arm time. MOR_Prob = morphine withdrawal + probiotic therapy (*n*=6); PLA_Prob = placebo + probiotic therapy (*n*=3); MOR_Water = morphine withdrawal + water control (*n*=6); PLA_Water = placebo + water control (*n*=3).

To explore potential mechanisms by which probiotic treatment during protracted morphine withdrawal was able to reduce the development of anxiety-like behavior in female mice, amygdala samples collected as part of the above experimental paradigm (*n* = 3–6) were submitted for bulk RNA-sequencing. Consistent with our previous findings, DEG analysis revealed that female mice withdrawn from morphine and treated with water during withdrawal (MOR_Water) exhibited a higher degree of upregulated- than downregulated genes compared to placebo-withdrawn mice treated with water (PLA_Water) (Figure 7(a)). Indeed, Ingenuity Pathway Analysis (IPA) revealed only significantly upregulated signaling pathways associated with the MOR_Water condition compared to PLA_Water (Figure 7(c)). Interestingly, morphine-withdrawn mice treated with probiotics (MOR_Prob) showed a greater degree of downregulated genes compared to the MOR_Water condition (Figure 7(a)). Accordingly, IPA showed only inhibited signaling pathways in the MOR_Prob condition compared to the MOR_Water condition (Figure 7(d)). The implication of these results is that morphine withdrawal broadly upregulates multiple signaling pathways that are in turn downregulated by probiotic therapy. Probiotic treatment in the placebo condition (PLA_Prob) was associated with relatively minor differential gene expression compared to the PLA_Water condition (Figure 7(a)), suggesting that the transcriptional alterations induced by probiotic treatment were specific to morphine withdrawal.

Several pathways identified by IPA as significantly inhibited in the morphine-probiotics condition compared to the morphine-water condition were noted as potentially relevant to the development of anxiety-like behavior, particularly serotonin receptor signaling. Counterintuitively, probiotic treatment during morphine withdrawal was also associated with inhibition of serotonin receptor signaling; significant downregulation of a total of nine genes was observed: *Htr6*, *Adcy5*, *Cacna1i*, *Pik3c2b*, *Rasd2*, *Shank3*, *Htr1b*, *Gng7*, and *Rhobtb2*. A multiple linear regression was employed to assess how expression of the nine downregulated genes on the serotonin signaling pathway influence time spent in the open arms of the elevated plus maze. The overall fit of the model (R^2^ = 0.993) was statistically significant (F(9,2) = 33.43, *p* = 0.0294), indicating that the model explains a significant portion of the variance in time spent in the open arms of the elevated plus maze (Figure 7(e)). Additionally, a correlation matrix was constructed to compare the expression of these genes with the time spent in the open arms of the elevated plus maze by the corresponding animal (Figure 7(f)). Spearman correlation analysis identified three genes that were significantly negatively correlated with time spent in the open arms of the maze: *Htr6* (*r* = −0.797, *p* = 0.003), *Pik3c2b* (*r* = −0.713, *p* = 0.012), and *Rhobtb2* (*r* = −0.720, *p* = 0.011).

### Probiotic treatment reverses withdrawal-induced transcriptional changes in monoaminergic signaling pathways

To investigate the molecular effects of prolonged opioid withdrawal and probiotic treatment on the brain, we examined transcriptional changes in the amygdala using a targeted RT^2^ PCR panel focused on genes related to dopamine, serotonin, and tryptophan signaling. Comparisons were made between animals undergoing morphine withdrawal with or without probiotics, and relevant controls, to determine how gut-targeted interventions may modulate neurochemical gene expression during abstinence. We focused on functional categories critical to neuromodulation – synthesis, transport, receptor expression, and metabolic enzymes – to identify consistent patterns across neurotransmitter systems.

**Figure 8.**
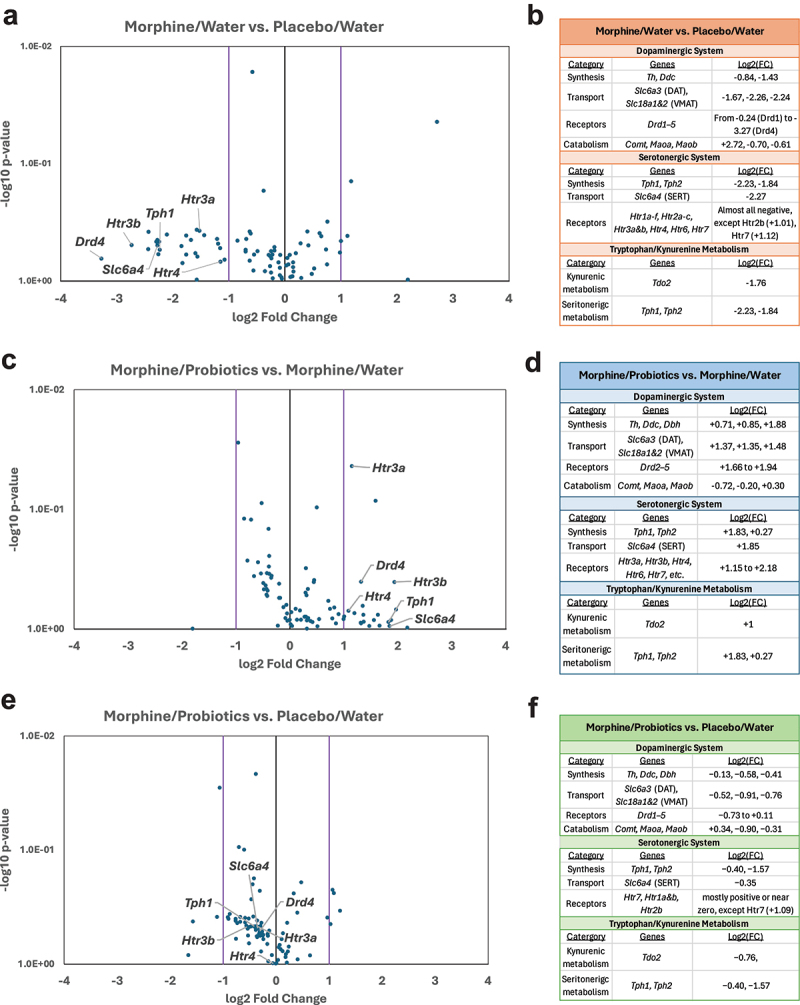
Neurotransmitter gene expression changes in the amygdala across morphine withdrawal and probiotic treatment. (a, c, e) volcano plots show differentially expressed genes between treatment groups, highlighting key dopamine, serotonin, and tryptophan-related transcripts. (b, d, f) pathway-level summaries show directionality and magnitude of gene expression changes across dopaminergic, serotonergic, and tryptophan-related pathways. Values shown represent log₂ Fold changes for representative genes in each category; *n*=3/group.

In animals undergoing protracted morphine withdrawal, we observed broad transcriptional suppression across both dopaminergic and serotonergic pathways (Figure 8(a)). Genes involved in synthesis (*Th, Tph1, Tph2*), transport (*Slc6a3, Slc6a4*), and receptor signaling (*Drd2–5, Htr3a&b, Htr4*) were consistently downregulated. Pathway-level analysis (Figure 8(b)) confirmed this suppression, with substantial negative log₂ fold changes observed across all monoaminergic categories. These findings reflect the disrupted neurochemical state associated with opioid withdrawal and align with previously reported behavioral and physiological phenotypes.

Following probiotic treatment, many of the same neurotransmitter-related genes showed marked upregulation, reversing the withdrawal- induced suppression (Figure 8(c)). In particular, transcripts encoding for serotonin receptors *(Htr3a, Htr3b, Htr4*), the serotonin transporter (*Slc6a4*), and enzymes involved in monoamine synthesis (*Tph1, Dbh*) demonstrated significant increases in expression. This pattern of transcriptional recovery was consistent across the major functional categories (Figure 8(d)), suggesting that probiotic treatment during withdrawal promotes restoration of neurochemical signaling pathways.

When comparing animals treated with probiotics to baseline controls, gene expression values remained moderately downregulated but were substantially closer to baseline than in the untreated withdrawal condition (Figure 8(e)). While certain transcripts such as *Tph2* and *Slc18a2* remained

suppressed, the overall pattern (Figure 8(f)) indicates partial normalization, with receptor and transporter expression showing milder deviations from placebo control levels. Together, these data demonstrate that probiotic treatment during opioid withdrawal can partially reverse withdrawal-associated transcriptional disruptions across multiple neurotransmitter systems in the amygdala. The changes observed at the gene expression level mirror known behavioral and physiological features of withdrawal and recovery, suggesting that probiotic-mediated modulation of the gut-brain axis may contribute to restoring neural homeostasis.

## Discussion

The development of anxiety symptoms is a common feature of protracted opioid withdrawal; however previous studies have not investigated the role of the gut microbiome in this behavior. In this study, we first show that protracted morphine withdrawal is associated with gut microbial dysbiosis and altered gut metabolites. Second, we provide evidence that morphine withdrawal-induced gut dysbiosis contributes to the development of anxiety-like behavior, which can be prevented by the administration of probiotics during withdrawal. Further, we propose a mechanism of altered amygdala serotonin receptor signaling that may mediate the interaction of the opioid-induced gut dysbiosis and anxiety-like behavior. These results advance our understanding of the gut microbiome during opioid withdrawal and consequently support the use of probiotic therapy for individuals discontinuing chronic opioid use.

16S rRNA sequencing data indicate gut dysbiosis in both female and male mice which persists into the protracted phase of morphine withdrawal. Specifically, there were significant shifts in beta diversity, indicating distinct differences in microbial composition between morphine-withdrawn mice and placebo controls. Interestingly, female mice demonstrated an increase in alpha diversity, contrasting with the decrease in alpha diversity previously associated with morphine dependence.^14^ This suggests the partial rebound of microbial diversity observed during the first 24 h of morphine withdrawal^17^ continued beyond the point of placebo controls over the following week. In both female and male mice, we showed an expansion of gut flora associated with inflammation and dysbiosis. In female mice, we observed an expansion of the phylum Proteobacteria, a proposed microbial signature of gut dysbiosis^62^ which is linked to inflammatory bowel disease (IBD)^63,64^ and chronic stress.^65^ Male mice withdrawn from chronic morphine treatment presented with an enrichment of Bacteroidota and a depletion of Firmicutes at the phylum level. A disturbance to the ratio of the abundance of these two phyla (F/B ratio) is indicative of gut dysbiosis.^66^ Specifically, the decreased F/B ratio observed here in males withdrawn from morphine is typically associated with inflammatory bowel disease.^66–68^ Both female and male mice also show a depletion of probiotic flora following morphine withdrawal. Female mice withdrawn from morphine showed a depletion of the *Ligilactobacillus* genus and the species *Bacteroides uniformis*. *Bacteroides uniformis*, a commercially available probiotic, is associated with metabolic and immune benefits^69,70^ and has even been termed a “psychobiotic” due to its role in serotonin metabolism and reduction of anxiety-like behaviors.^71^ Male mice withdrawn from morphine showed a notable depletion of Bifidobacterium, a genus widely recognized for its probiotic properties.^72^ Bifidobacteria inhibit LPS-induced NF-κB activation,^73^ prevent pathogen expansion,^74^ and have been established to reduce anxiety in both mice^75^ and humans.^35^ Overall, protracted morphine withdrawal is associated with an expansion of dysbiotic gut flora along with a depletion of beneficial bacterial groups that are associated with anxiety like behavior.

To better understand how morphine withdrawal-induced gut dysbiosis may influence host physiology and behavior, we conducted untargeted metabolomic analysis of colon contents. Our metabolomic analysis revealed that morphine withdrawal induces a distinct shift in gut metabolite composition, with significant alterations in pathways related to neurotransmitter regulation, most notably tryptophan metabolism. Elevated levels of kynurenic acid alongside increased 5-HIAA and reduced serotonin sulfate suggest that tryptophan is preferentially metabolized through the kynurenine pathway at the expense of central serotonin synthesis. This diversion may contribute to the serotonergic imbalance implicated in withdrawal-induced anxiety-like behavior. These findings provide a potential mechanistic link between gut dysbiosis and behavioral outcomes, offering further support for a microbiota-gut-brain axis disrupted by opioid withdrawal.

Using fecal microbiota transplantation (FMT), we demonstrated that gut dysbiosis associated with morphine withdrawal is sufficient to induce anxiety-like behavior in treatment-naive mice. This finding supports a causal role for the gut microbiome in the development of negative affective, such as anxiety-like behavior, during morphine withdrawal. FMT includes the transfer of bacteria as well as their metabolites,^76^ which may also impact behavior.^77^ Bacterial metabolites include bidirectional modulators of neuroinflammation, neuroactive mediators, neurotransmitter receptor agonists, and other products capable of orchestrating a speedy behavioral response.^78^ Relevantly, Thomaz et al. (2021) demonstrated that FMT could attenuate opioid withdrawal symptoms in morphine-dependent mice without inducing stable changes to gut microbiota composition, suggesting that transient microbial activity or metabolite transfer may be sufficient to alter behavioral outcomes.^18^ Given the short treatment duration and relatively brief time before behavioral assessment in this experiment, the effect of FMT is likely driven by microbial metabolites present in the transplant material, rather than the eventual engraftment of bacterial groups. This interpretation is supported by our untargeted metabolomic analysis, which revealed distinct metabolic profiles between donor groups. While our study focused on bacterial community structure and function, the FMT solution likely also contained bacteriophages and other components of the gut virome, which may have contributed to the observed behavioral effects by modulating microbial dynamics or host signaling pathways.^79^ The role of the gut virome in shaping neurobehavioral outcomes, particularly in the context of opioid withdrawal, remains a promising direction for future investigation.

Building on our observation of depleted probiotic bacterial groups associated with morphine withdrawal, we investigated whether probiotic supplementation during protracted morphine withdrawal could mitigate the induction of anxiety-like behavior. To enhance clinical relevance, we selected a commercially available probiotic blend (VSL#3) for testing. Morphine-withdrawn mice treated with VSL#3 exhibited reduced anxiety-like behavior compared to morphine-withdrawn mice given a water gavage, suggesting an anxiolytic effect of the probiotic treatment. Probiotic therapy had no effect on placebo-treated mice, indicating that the anxiolytic effect was specific to morphine withdrawal. We propose that VSL#3 treatment countered the dysbiotic expansion observed via 16S sequencing by supplementing the depleted probiotic groups, potentially preventing the onset of anxiety-like behavior. These results support the feasibility of probiotic therapy as a potential adjunct treatment in opioid detox settings to alleviate some of the negative emotional effects of opioid withdrawal.

To identify mechanisms by which gut dysbiosis following protracted morphine withdrawal may contribute to anxiety-like behavior, we conducted bulk RNA-sequencing on amygdala samples from mice withdrawn from either morphine or placebo. The amygdala is known to influence hedonic processing during protracted morphine withdrawal^80^ and to modulate the development of withdrawal-associated anxiety.^43,81^ Ingenuity Pathway Analysis (IPA) of the RNA-sequencing data revealed significant downregulation of serotonin signaling in morphine-withdrawn female mice, suggesting a possible link to anxiety-like behavior. Within this pathway, serotonin receptors *Htr2c* and *Htr5b* were notably upregulated. Prior studies indicate that *Htr2c* knockout can reduce anxiety-like behavior by dampening amygdala signaling,^82^ highlighting a potential role of *Htr2c* upregulation in anxiety during withdrawal. Interestingly, despite the overall downregulation of serotonin signaling, several pathway genes, including *Htr6*, were paradoxically upregulated.

To further investigate how the gut microbiome may impact anxiety-like behavior during morphine withdrawal, we conducted additional bulk RNA-sequencing on amygdala samples, this time collected from mice under the probiotic therapy paradigm. IPA showed a trend of predominantly upregulated pathways in the morphine withdrawal condition without probiotics, whereas probiotic treatment led to a generalized downregulation of these pathways. Most notably, serotonin signaling was downregulated in the morphine-probiotic condition compared to the morphine-water control. While this might seem counterintuitive, it is contextualized by examination of the genes that drive the expression of this pathway. For instance, *Htr1b* expression was reduced in the morphine-probiotic condition relative to morphine-water, aligning with evidence that 5-HT1B receptor antagonists can have antidepressive effects, while agonists may induce depressive-like behavior.^83,84^ Expression of *Htr6* was also lower in the morphine-probiotic condition, mirroring previous RNA-sequencing results that found elevated *Htr6* expression associated with morphine withdrawal. Intriguingly, both agonists and antagonists of the 5-HT6 receptor have shown anxiolytic effects,^85^ emphasizing the complex interplay between serotonin signaling and anxiety-related behaviors.

Of the nine serotonin signaling genes downregulated in the morphine-probiotic group, six had been upregulated in our initial RNA-sequencing analysis of morphine-withdrawn females (*Htr6*, *Adcy5*, *Cacna1i*, *Pik3c2b*, *Rasd2*, and *Shank3*). Expression of these genes also strongly predicted anxiety-like behavior in the elevated plus maze, as confirmed by linear regression analysis. Together, these findings suggest that morphine withdrawal upregulates specific serotonergic genes in female mice, which are subsequently downregulated following probiotic therapy. This pattern highlights a potential mechanism by which probiotic therapy may modulate the gut-brain axis to prevent anxiety-like behavior during morphine withdrawal.

Sex-specific differences in behavior, microbiota composition, and gene expression were observed across multiple datasets in this study. These findings are consistent with prior literature indicating that male and female rodents may exhibit divergent neurobiological and behavioral responses to opioid exposure and withdrawal.^86^ Importantly, our untargeted metabolomic analysis revealed alterations in sex hormone-related metabolites, including estradiol and testosterone derivatives, as well as corticosterone, a key stress hormone. These findings suggest that morphine withdrawal may impact endocrine signaling pathways in females, potentially contributing to the observed disparities in anxiety-like behavior and transcriptional profiles. The gut microbiota is known to influence- and be influenced by-host sex hormone levels, raising the possibility that microbiome-endocrine interactions may amplify sex-specific responses during opioid withdrawal. Future studies with male mice will be essential to further dissect these mechanisms.

This study has several limitations. First, while placebo-treated animals served as the standard control for surgical and handling variables, the absence of a surgery-naive group prevents assessment of any independent effects of pellet implantation. Second, we did not perform microbiome or metabolomic profiling in FMT recipients, limiting our ability to confirm engraftment or directly link transferred metabolites to behavioral effects. Third, we did not conduct metabolomic analysis following probiotic treatment, so mechanistic interpretation of the probiotic’s effects is limited to behavioral and transcriptional outcomes. Future studies incorporating these additional analyses will help further clarify microbiota-brain interactions in opioid withdrawal.

These findings provide compelling evidence that protracted morphine withdrawal induces gut microbial dysbiosis, which in turn contributes to the development of anxiety-like behavior. By combining 16S rRNA sequencing, untargeted metabolomic analysis, fecal microbiota transplantation, and probiotic therapy, we demonstrate both the causality of gut dysbiosis in the onset of anxiety-like behaviors and the potential of probiotic intervention to mitigate these effects. Our results further suggest a complex interaction between gut microbiota and central serotonin signaling in the amygdala, revealing alterations in serotonergic pathways that may underlie the behavioral outcomes observed during opioid withdrawal. Specifically, probiotic supplementation appears to restore balance to the gut microbiome, downregulate specific serotonin receptors, and reduce anxiety-like behavior in morphine-withdrawn mice. These findings not only support the gut-brain axis as a key mediator of opioid withdrawal symptoms but also propose probiotic therapy as a promising adjunct treatment for managing the emotional and psychological challenges associated with opioid detoxification. Future studies should explore the clinical implications of these results, particularly in the context of human opioid withdrawal, to determine the feasibility of implementing probiotic-based therapies in opioid recovery programs.

## Supplementary Material

Supplementary Figures.docx

## Data Availability

The data that support the findings of this study are openly available in the Harvard Dataverse at https://dataverse.harvard.edu/dataverse/GMBANX. (https://doi.org/10.7910/DVN/ZXEWRE., https://doi.org/10.7910/DVN/YCP2HH., https://doi.org/10.7910/DVN/NL1Y3I., https://doi.org/10.7910/DVN/5XJKQC., https://doi.org/10.7910/DVN/RLUNWT., https://doi.org/10.7910/DVN/K07FX0.) The generative AI ChatGPT-4-turbo was employed to improve grammar and flow of introduction and discussion sections.

## References

[1] Strang J, Volkow ND, Degenhardt L, Hickman M, Johnson K, Koob GF, Marshall BDL, Tyndall M, Walsh SL. Opioid use disorder. Nat Rev Dis Primers. 2020;6(1):3. doi: 10.1038/s41572-019-0137-5. 31919349

[2] Weiss RD, Potter JS, Griffin ML, McHugh RK, Haller D, Jacobs P, Gardin J, Fischer D, Rosen KD. Reasons for opioid use among patients with dependence on prescription opioids: the role of chronic pain. J Subst Abuse Treat. 2014;47(2):140–26. doi: 10.1016/j.jsat.2014.03.004. 24814051 PMC4074437

[3] Pergolizzi JV Jr, Rosenblatt M, Mariano DJ, LeQuang JA. Pain Management 9 2. ; 2019 Clinical considerations about opioid withdrawal syndrome. p. 111–113 doi: 10.2217/pmt-2018-0088. 30806586

[4] Pergolizzi JV Jr, Raffa RB, Rosenblatt MH. Opioid withdrawal symptoms, a consequence of chronic opioid use and opioid use disorder: current understanding and approaches to management. J Clin Pharm Ther. 2020;45(5):892–903. doi: 10.1111/jcpt.13114. 31986228

[5] Bruneau A, Frimerman L, Verner M, Sirois A, Fournier C, Scott K, Perez J, Shir Y, Martel MO. Day-to-day opioid withdrawal symptoms, psychological distress, and opioid craving in patients with chronic pain prescribed opioid therapy. Drug Alcohol depend. Drug Alcohol Depen. 2021;225:108787. doi: 10.1016/j.drugalcdep.2021.108787. 34091157

[6] Moradinazar M, Farnia V, Alikhani M, Asadi A, Marzbani B, Najafi F. The effects of anxiety on relapse of patients with opioid use disorders under methadone maintenance treatment: control of the confounding variables. J Subst Use. 2020;25(1):34–39. doi: 10.1080/14659891.2019.1659868.

[7] Baxley C, Weinstock J, Lustman PJ, Garner AA. The influence of anxiety sensitivity on opioid use disorder treatment outcomes. Exp Clin Psychopharmacol. 2019;27(1):64. doi: 10.1037/pha0000215. 30080059

[8] Ferri M, Finlayson AJR, Wang L, Martin PR. Predictive factors for relapse in patients on buprenorphine maintenance. Am J Addictions. 2014;23(1):62–67. doi: 10.1111/j.1521-0391.2013.12074.x. PMC3922612 24313243

[9] Rogers AH, Zvolensky MJ, Ditre JW, Buckner JD, Asmundson GJG. Association of opioid misuse with anxiety and depression: a systematic review of the literature. Clin Psychol Rev. 2021;84:101978. doi: 10.1016/j.cpr.2021.101978. 33515811

[10] Zhang L, Roy S. Opioid modulation of the gut–brain axis in opioid-associated comorbidities. Cold Spring harbor perspectives in medicine. Cold Spring Harb Perspect Med. 2021;11(9):a040485. doi: 10.1101/cshperspect.a040485. 32816876 PMC8415294

[11] Herlihy B, Roy S. Gut-microbiome implications in opioid use disorder and related behaviors. Adv Drug Alcohol Res. 2022;2:10311. doi: 10.3389/adar.2022.10311. 38390617 PMC10880781

[12] Wang F, Roy S. Gut homeostasis, microbial dysbiosis, and opioids. Toxicologic Pathol. 2017;45(1):150–156. doi: 10.1177/0192623316679898. PMC8607522 27895265

[13] Jalodia R, Abu YF, Oppenheimer MR, Herlihy B, Meng J, Chupikova I, Tao J, Ghosh N, Dutta RK, Kolli U, et al. Opioid use, gut dysbiosis, inflammation, and the nervous system. J Neuroimmune Pharmacol. 2022;17(1–2):1–18. doi: 10.1007/s11481-021-10046-z. 34993905 PMC10276355

[14] Wang F, Meng J, Zhang L, Johnson T, Chen C, Roy S. Morphine induces changes in the gut microbiome and metabolome in a morphine dependence model. Sci Rep. 2018;8(1):3596. doi: 10.1038/s41598-018-21915-8. 29483538 PMC5827657

[15] Meng J, Yu H, Ma J, Wang J, Banerjee S, Charboneau R, Barke RA, Roy S. Morphine induces bacterial translocation in mice by compromising intestinal barrier function in a TLR-dependent manner. PLOS ONE. 2013;8(1):e54040. doi: 10.1371/journal.pone.0054040. 23349783 PMC3548814

[16] Zhang L, Meng J, Ban Y, Jalodia R, Chupikova I, Fernandez I, Brito N, Sharma U, Abreu MT, Ramakrishnan S, et al. Morphine tolerance is attenuated in germfree mice and reversed by probiotics, implicating the role of gut microbiome. Proc Natl Acad Sci USA. 2019;116(27):13523–13532. doi: 10.1073/pnas.1901182116. 31209039 PMC6613141

[17] Truitt B, Venigalla G, Singh P, Singh S, Tao J, Chupikova I, Roy S. The gut microbiome contributes to somatic morphine withdrawal behavior and implicates a TLR2 mediated mechanism. Gut Microbes. 2023;15(1):2242610. doi: 10.1080/19490976.2023.2242610. 37589387 PMC10438851

[18] Thomaz AC, Iyer V, Woodward TJ, Hohmann AG. Fecal microbiota transplantation and antibiotic treatment attenuate naloxone-precipitated opioid withdrawal in morphine-dependent mice. Exp Neurol. 2021;343:113787. doi: 10.1016/j.expneurol.2021.113787. 34153321 PMC8477666

[19] Foster JA, Neufeld K-AM. Gut–brain axis: how the microbiome influences anxiety and depression. Trends in neurosciences. Trends Neurosciences. 2013;36(5):305–312. doi: 10.1016/j.tins.2013.01.005. 23384445

[20] Lee Y, Kim Y-K. Understanding the connection between the gut–brain axis and stress/anxiety disorders. Curr Psychiatry Rep. 2021;23(5):1–7. doi: 10.1007/s11920-021-01235-x. 33712947

[21] Popa S-L, Dumitrascu DL. Anxiety and IBS revisited: ten years later. Clujul Med. 2015;88(3):253. doi: 10.15386/cjmed-495. 26609253 PMC4632879

[22] Chen Y-H, Bai J, Wu D, Yu S-F, Qiang X-L, Bai H, Wang H-N, Peng Z-W. Association between fecal microbiota and generalized anxiety disorder: severity and early treatment response. J Affect Disord. 2019;259:56–66. doi: 10.1016/j.jad.2019.08.014. 31437702

[23] Kim S-Y, Woo S-Y, Raza S, Ho D, Jeon SW, Chang Y, Ryu S, Kim H-L, Kim H-N. Association between gut microbiota and anxiety symptoms: a large population-based study examining sex differences. J Affect Disord. 2023;333:21–29. doi: 10.1016/j.jad.2023.04.003. 37031878

[24] Slyepchenko A, Carvalho A, Cha D, Kasper S, McIntyre R. Gut emotions-mechanisms of action of probiotics as novel therapeutic targets for depression and anxiety disorders. CNS & Neurological disorders-Drug targets-Cns & Neurological Disorders). 2014;13(10):1770–1786. doi: 10.2174/1871527313666141130205242. 25470391

[25] Molavi N, Rasouli-Azad M, Mirzaei H, Matini AH, Banafshe HR, Valiollahzadeh M, Hassanzadeh M, Saghazade AR, Abbaszadeh-Mashkani S, Mamsharifi P, et al. The effects of probiotic supplementation on opioid-related disorder in patients under methadone maintenance treatment programs. International Journal Of Clinical practice int J Clin Pract. 2022;2022(1):1206914. doi: 10.1155/2022/1206914. PMC9159114 35685534

[26] Luczynski P, mcvey Neufeld K-A, Oriach CS, Clarke G, Dinan TG, Cryan JF. Growing up in a bubble: using germ-free animals to assess the influence of the gut microbiota on brain and behavior. Int J Neuropsychopharmacol. 2016;19(8):yw020. doi: 10.1093/ijnp/pyw020. PMC5006193 26912607

[27] Neufeld K, Kang N, Bienenstock J, Foster JA. Reduced anxiety‐like behavior and central neurochemical change in germ‐free mice. Neurogastroenterology Motil. 2011;23(3):255–e119. doi: 10.1111/j.1365-2982.2010.01620.x. 21054680

[28] Roussin L, Gry E, Macaron M, Ribes S, Monnoye M, Douard V, Naudon L, Rabot S. Microbiota influence on behavior: integrative analysis of serotonin metabolism and behavioral profile in germ-free mice. The FASEB J. 2024;38(11):e23648. doi: 10.1096/fj.202400334R. 38822661 PMC12086753

[29] Zierer J, Jackson MA, Kastenmüller G, Mangino M, Long T, Telenti A, Mohney RP, Small KS, Bell JT, Steves CJ, et al. The fecal metabolome as a functional readout of the gut microbiome. Nat Genet. 2018;50(6):790–795. doi: 10.1038/s41588-018-0135-7. 29808030 PMC6104805

[30] O’Riordan KJ, Collins MK, Moloney GM, Knox EG, Aburto MR, Fülling C, Morley SJ, Clarke G, Schellekens H, Cryan JF, et al. Short chain fatty acids: microbial metabolites for gut-brain axis signalling. Mol And Cellular Endocrinol. 2022;546:111572. doi: 10.1016/j.mce.2022.111572. 35066114

[31] Monteiro-Cardoso VF, Corlianò M, Singaraja RR. Bile acids: a communication channel in the gut-brain axis. NeuroMol Med. 2021;23(1):99–117. doi: 10.1007/s12017-020-08625-z. 33085065

[32] Bosi A, Banfi D, Bistoletti M, Giaroni C, Baj A. Tryptophan metabolites along the microbiota-gut-brain axis: an interkingdom communication System influencing the gut in health and disease. Int J Multiling Tryptophan Research. 2020;13:1178646920928984. doi: 10.1177/1178646920928984. PMC7290275 32577079

[33] D’Mello C, Ronaghan N, Zaheer R, Dicay M, Le T, MacNaughton WK, Surrette MG, Swain MG. Probiotics improve inflammation-associated sickness behavior by altering communication between the peripheral immune system and the brain. J Neurosci. 2015;35(30):10821–10830. doi: 10.1523/JNEUROSCI.0575-15.2015. 26224864 PMC6605112

[34] Li N, Wang Q, Wang Y, Sun A, Lin Y, Jin Y, Li X. Oral probiotics ameliorate the behavioral deficits induced by chronic mild stress in mice via the gut microbiota-inflammation axis. Frontiers in behavioral neuroscience. Front Behavioral Neurosci. 2018;12:266. doi: 10.3389/fnbeh.2018.00266. PMC6232506 30459574

[35] Ma T, Jin H, Kwok L-Y, Sun Z, Liong M-T, Zhang H. Probiotic consumption relieved human stress and anxiety symptoms possibly via modulating the neuroactive potential of the gut microbiota. Neurobiol Stress. 2021;14:100294. doi: 10.1016/j.ynstr.2021.100294. 33511258 PMC7816019

[36] Wall R, Cryan JF, Ross RP, Fitzgerald GF, Dinan TG, Stanton C. Bacterial neuroactive compounds produced by psychobiotics. Microbial endocrinology: the microbiota-gut-brain axis in health and disease Adv Exp Med Biol. 817. 2014; 221–239 doi:10.1007/978-1-4939-0897-4_10. 24997036

[37] Ozdemir D, Allain F, Kieffer BL, Darcq E. Advances in the characterization of negative affect caused by acute and protracted opioid withdrawal using animal models. Neuropharmacology. 2023;232:109524. doi: 10.1016/j.neuropharm.2023.109524. 37003572 PMC10844657

[38] Zanos P, Georgiou P, Wright SR, Hourani SM, Kitchen I, Winsky-Sommerer R, Bailey A. The oxytocin analogue carbetocin prevents emotional impairment and stress-induced reinstatement of opioid-seeking in morphine-abstinent mice. Neuropsychopharmacology. 2014;39(4):855–865. doi: 10.1038/npp.2013.285. 24129263 PMC3924520

[39] Zanos P, Georgiou P, Gonzalez LR, Hourani S, Chen Y, Kitchen I, Kieffer BL, Winsky-Sommerer R, Bailey A. Emotional impairment and persistent upregulation of mGlu5 receptor following morphine abstinence: implications of an mGlu5-MOPr interaction. Int J Neuropsychopharmacol. 2016;19(7):pyw011. doi: 10.1093/ijnp/pyw011. 26861145 PMC4966274

[40] Ma H, Wang N, Wang X, Jia M, Li Y, Cui C. Wnt7a in mouse insular cortex contributes to anxiety-like behavior during protracted abstinence from morphine. Neuroscience. 2018;394:164–176. doi: 10.1016/j.neuroscience.2018.10.032. 30367944

[41] Bravo IM, Luster BR, Flanigan ME, Perez PJ, Cogan ES, Schmidt KT, McElligott ZA. Divergent behavioral responses in protracted opioid withdrawal in male and female C57BL/6J mice. Eur J Neurosci. 2020;51(3):742–754. doi: 10.1111/ejn.14580. 31544297 PMC7069788

[42] Lutz P-E, Ayranci G, Chu-Sin-Chung P, Matifas A, Koebel P, Filliol D, Befort K, Ouagazzal A-M, Kieffer BL. Distinct mu, delta, and kappa opioid receptor mechanisms underlie low sociability and depressive-like behaviors during heroin abstinence. Neuropsychopharmacology. 2014;39(11):2694–2705. doi: 10.1038/npp.2014.126. 24874714 PMC4207349

[43] Deji C, Yan P, Ji Y, Yan X, Feng Y, Liu J, Liu Y, Wei S, Zhu Y, Lai J, et al. The basolateral amygdala to ventral hippocampus circuit controls anxiety-like behaviors induced by morphine withdrawal. Front Cell Neurosci. 2022;16:894886. doi: 10.3389/fncel.2022.894886. 35726232 PMC9205755

[44] Kaplan GB, Thompson BL. Neuroplasticity of the extended amygdala in opioid withdrawal and prolonged opioid abstinence. Front Pharmacol. 2023;14:1253736. doi: 10.3389/fphar.2023.1253736. 38044942 PMC10690374

[45] Cabral A, Ruggiero RN, Nobre MJ, Brandão ML, Castilho VM. GABA and opioid mechanisms of the central amygdala underlie the withdrawal-potentiated startle from acute morphine. Prog Neuropsychopharmacol Biol Psychiatry. 2009;33(2):334–344. doi: 10.1016/j.pnpbp.2008.12.012. 19150477

[46] Hofford RS, Hodgson SR, Roberts KW, Bryant CD, Evans CJ, Eitan S. Extracellular signal-regulated kinase activation in the amygdala mediates elevated plus maze behavior during opioid withdrawal. Behavioural Pharmacol. 2009;20(7):576–583. doi: 10.1097/FBP.0b013e32832ec57e. PMC4494789 19738463

[47] Asan E, Steinke M, Lesch K-P. Serotonergic innervation of the amygdala: targets, receptors, and implications for stress and anxiety. Histochem And Cell Biol. 2013;139(6):785–813. doi: 10.1007/s00418-013-1081-1. 23494464

[48] Jia M, Meng F, Smerin SE, Xing G, Zhang L, Su DM, Benedek D, Ursano R, Su YA, Li H, et al. Biomarkers in an animal model for revealing neural, hematologic, and behavioral correlates of PTSD. J Visualized Experiments: Jove. 2012;(68):68. doi: 10.3791/3361-v. PMC3490307 23093202

[49] Spijker S, Li KW, Editor. Dissection of rodent brain regions, in *Neuroproteomics*, Totowa (NJ): Humana Press; 2011. p. 13–26.

[50] Callahan BJ, McMurdie PJ, Rosen MJ, Han AW, Johnson AJA, Holmes SP. DADA2: high-resolution sample inference from Illumina amplicon data. Nat Methods. 2016;13(7):581. doi: 10.1038/nmeth.3869. 27214047 PMC4927377

[51] Wang Q, Garrity GM, Tiedje JM, Cole JR. Naive Bayesian classifier for rapid assignment of rRNA sequences into the new bacterial taxonomy. Appl Environ Microb. 2007;73(16):5261–5267. doi: 10.1128/AEM.00062-07. PMC1950982 17586664

[52] Yilmaz P, Parfrey LW, Yarza P, Gerken J, Pruesse E, Quast C, Schweer T, Peplies J, Ludwig W, Glöckner FO, et al. The SILVA and “all-species living tree project (LTP)” taxonomic frameworks. Nucleic Acids Res. 2014;42(D1):D643–D648. doi: 10.1093/nar/gkt1209. 24293649 PMC3965112

[53] Chong J, Liu P, Zhou G, Xia J. Using MicrobiomeAnalyst for comprehensive statistical, functional, and meta-analysis of microbiome data. Nat Protoc. 2020;15(3):799–821. doi: 10.1038/s41596-019-0264-1. 31942082

[54] Segata N, Izard J, Waldron L, Gevers D, Miropolsky L, Garrett WS, Huttenhower C. Metagenomic biomarker discovery and explanation. Genome Biology. 2011;12(6):1–18. doi: 10.1186/gb-2011-12-6-r60. PMC3218848 21702898

[55] Pang Z, Lu Y, Zhou G, Hui F, Xu L, Viau C, Spigelman A, MacDonald P, Wishart D, Li S, et al. MetaboAnalyst 6.0: towards a unified platform for metabolomics data processing, analysis and interpretation. Nucleic Acids Res. 2024;52(W1):W398–W406. doi: 10.1093/nar/gkae253. 38587201 PMC11223798

[56] Pertea M, Kim D, Pertea GM, Leek JT, Salzberg SL. Transcript-level expression analysis of RNA-seq experiments with hisat, StringTie and Ballgown. Nat Protoc. 2016;11(9):1650–1667. doi: 10.1038/nprot.2016.095. 27560171 PMC5032908

[57] Liao Y, Smyth GK, Shi W. featureCounts: an efficient general purpose program for assigning sequence reads to genomic features. Bioinformatics. 2014;30(7):923–930. doi: 10.1093/bioinformatics/btt656. 24227677

[58] Love MI, Huber W, Anders S. Moderated estimation of Fold change and dispersion for RNA-seq data with DESeq2. Genome Biology. 2014;15(12):1–21. doi: 10.1186/s13059-014-0550-8. PMC4302049 25516281

[59] Agus A, Planchais J, Sokol H. Gut microbiota regulation of tryptophan metabolism in health and disease. Cell Host & Microbe. 2018;23(6):716–724. doi: 10.1016/j.chom.2018.05.003. 29902437

[60] Cowan CSM, Hoban AE, Ventura‐Silva AP, Dinan TG, Clarke G, Cryan JF. Gutsy moves: the amygdala as a critical node in microbiota to brain signaling. BioEssays. 2018;40(1):1700172. doi: 10.1002/bies.201700172. 29148060

[61] Graeff FG. On serotonin and experimental anxiety. Psychopharmacology. 2002;163(3–4):467–476. doi: 10.1007/s00213-002-1112-4. 12373447

[62] Shin N-R, Whon TW, Bae J-W. Proteobacteria: microbial signature of dysbiosis in gut microbiota. Trends in biotechnology. Trends Biotechnol. 2015;33(9):496–503. doi: 10.1016/j.tibtech.2015.06.011. 26210164

[63] Rizzatti G, Lopetuso LR, Gibiino G, Binda C, Gasbarrini A. Proteobacteria: a common factor in human diseases. biomed Res Int. 2017;2017(1):1–7. doi: 10.1155/2017/9351507. PMC5688358 29230419

[64] Mukhopadhya I, Hansen R, El-Omar EM, Hold GL. IBD—what role do Proteobacteria play? Nature reviews gastroenterology & hepatology. Nat Rev Gastroenterol Hepatol. 2012;9(4):219–230. doi: 10.1038/nrgastro.2012.14. 22349170

[65] Langgartner D, Peterlik D, Foertsch S, Füchsl AM, Brokmann P, Flor PJ, Shen Z, Fox JG, Uschold-Schmidt N, Lowry CA, et al. Individual differences in stress vulnerability: the role of gut pathobionts in stress-induced colitis. Brain Behav Immun. 2017;64:23–32. doi: 10.1016/j.bbi.2016.12.019. 28012830

[66] Stojanov S, Berlec A, Štrukelj B. The influence of probiotics on the firmicutes/bacteroidetes ratio in the treatment of obesity and inflammatory bowel disease. Microorganisms. 2020;8(11):1715. doi: 10.3390/microorganisms8111715. 33139627 PMC7692443

[67] Cundra L, Saadeh M, Vallabhaneni M, Houston K, D’Souza S, Johnson DA. Dietary modulation of the gut microbiome in inflammatory bowel disease. Recent Prog In Nutr. 2022;2(3):1–24. doi: 10.21926/rpn.2203019.

[68] Wong W-Y, Chan BD, Leung T-W, Chen M, Tai WCS. Beneficial and anti-inflammatory effects of formulated prebiotics, probiotics, and synbiotics in normal and acute colitis mice. J Funct Foods. 2022;88:104871. doi: 10.1016/j.jff.2021.104871.

[69] Gauffin Cano P, Santacruz A, Moya Á, Sanz Y. Bacteroides uniformis CECT 7771 ameliorates metabolic and immunological dysfunction in mice with high-fat-diet induced obesity. PLOS ONE. 2012;7(7):e41079. doi: 10.1371/journal.pone.0041079. 22844426 PMC3406031

[70] Agustí A, Campillo I, Balzano T, Benítez-Páez A, López-Almela I, Romaní-Pérez M, Forteza J, Felipo V, Avena NM, Sanz Y, et al. Bacteroides uniformis CECT 7771 modulates the brain reward response to reduce binge eating and anxiety-like behavior in rat. Mol Neurobiol. 2021;58(10):4959–4979. doi: 10.1007/s12035-021-02462-2. 34228269 PMC8497301

[71] Hao Z, Meng C, Li L, Feng S, Zhu Y, Yang J, Han L, Sun L, Lv W, Figeys D, et al. Positive mood-related gut microbiota in a long-term closed environment: a multiomics study based on the “lunar palace 365” experiment. Microbiome. 2023;11(1):88. doi: 10.1186/s40168-023-01506-0. 37095530 PMC10124008

[72] Chen J, Chen X, Ho CL. Recent development of probiotic bifidobacteria for treating human diseases. Frontiers in bioengineering and biotechnology. 2021;9:770248. doi: 10.3389/fbioe.2021.770248. PMC8727868 35004640

[73] Riedel CU, Foata F, Philippe D, Adolfsson O, Eikmanns BJ, Blum S. Anti-inflammatory effects of bifidobacteria by inhibition of LPS-induced NF-κB activation. World J Gastroenterol: WJG. 2006;12(23):3729. doi: 10.3748/wjg.v12.i23.3729. 16773690 PMC4087466

[74] Sharma M, Wasan A, Sharma R. Recent developments in probiotics: an emphasis on Bifidobacterium. Food Biosci. 2021;41:100993. doi: 10.1016/j.fbio.2021.100993.

[75] Savignac H, Tramullas M, Kiely B, Dinan TG, Cryan JF. Bifidobacteria modulate cognitive processes in an anxious mouse strain. Behavioural Brain Res. 2015;287:59–72. doi: 10.1016/j.bbr.2015.02.044. 25794930

[76] Bokoliya SC, Dorsett Y, Panier H, Zhou Y. Procedures for fecal microbiota transplantation in murine microbiome studies. Frontiers in cellular and infection microbiology. Front Cell Infect Microbiol. 2021;11:711055. doi: 10.3389/fcimb.2021.711055. 34621688 PMC8490673

[77] Averina OV, Zorkina YA, Yunes RA, Kovtun AS, Ushakova VM, Morozova AY, Kostyuk GP, Danilenko VN, Chekhonin VP. Bacterial metabolites of human gut microbiota correlating with depression. Int J Mol Sci. 2020;21(23):9234. doi: 10.3390/ijms21239234. 33287416 PMC7730936

[78] Ahmed H, Leyrolle Q, Koistinen V, Kärkkäinen O, Layé S, Delzenne N, Hanhineva K. Microbiota-derived metabolites as drivers of gut–brain communication. Gut Microbes. 2022;14(1):2102878. doi: 10.1080/19490976.2022.2102878. 35903003 PMC9341364

[79] Ritz NL, Draper LA, Bastiaanssen TFS, Turkington CJR, Peterson VL, van de Wouw M, Vlckova K, Fülling C, Guzzetta KE, Burokas A, et al. The gut virome is associated with stress-induced changes in behaviour and immune responses in mice. Nat Microbiol. 2024;9(2):359–376. doi: 10.1038/s41564-023-01564-y. 38316929 PMC10847049

[80] Harris GC, Aston-Jones G. Activation in extended amygdala corresponds to altered hedonic processing during protracted morphine withdrawal. Behavioural Brain Res. 2007;176(2):251–258. doi: 10.1016/j.bbr.2006.10.012. PMC1809796 17123639

[81] Rothwell PE, Thomas MJ, Gewirtz JC. Distinct profiles of anxiety and dysphoria during spontaneous withdrawal from acute morphine exposure. Neuropsychopharmacology. 2009;34(10):2285–2295. doi: 10.1038/npp.2009.56. 19494807 PMC2726902

[82] Heisler LK, Zhou L, Bajwa P, Hsu J, Tecott LH. Serotonin 5-HT2C receptors regulate anxiety-like behavior. Genes Brain Behav. 2007;6(5):491–496. doi: 10.1111/j.1601-183X.2007.00316.x. 17451451

[83] Tatarczynska E, Kłdzińska A, Stachowicz K, Chojnacka-Wójcik E. P. 1.054 effect of combined administration of 5-HT 1 a or 5-HT 1 B receptor antagonists and antidepressant drugs in the forced swimming test in rats. Eur Neuropsychopharmacol. 2003;2003(13):S197–S198. doi: 10.1016/S0924-977X(03)91765-2.

[84] Chenu F, David DJP, Leroux-Nicollet I, Le Maître E, Gardier AM, Bourin M. Serotonin1B heteroreceptor activation induces an antidepressant-like effect in mice with an alteration of the serotonergic system. J Psychiatr Neurosci. 2008;33(6):541–550. PMC2575758 18982177

[85] Wesołowska A, Nikiforuk A. Effects of the brain-penetrant and selective 5-HT6 receptor antagonist SB-399885 in animal models of anxiety and depression. Neuropharmacology. 2007;52(5):1274–1283. doi: 10.1016/j.neuropharm.2007.01.007. 17320917

[86] Hodgson SR, Hofford RS, Roberts KW, Eitan D, Wellman PJ, Eitan S. Sex differences in affective response to opioid withdrawal during adolescence. J Psychopharmacol (oxf). 2010;24(9):1411–1417. doi: 10.1177/0269881109106976. PMC4494787 19939877

