# Supplementary material for: Anxiety-like behavior during protracted morphine withdrawal is driven by gut microbial dysbiosis and attenuated with probiotic treatment
Source: Gut Microbes. 2025 Jun 15;17(1):2517838. doi: 10.1080/19490976.2025.2517838 (PMC12169037; doi:10.1080/19490976.2025.2517838)
Supplement: Supplementary Figures.docx [file KGMI_A_2517838_SM1652.docx]

**Supplementary Figure 2.** Neither morphine withdrawal nor probiotic treatment was associated with behavioral differences in the open field test. (A) Percent activity in the center of the open field test (B) Total activity measured by number of beam breaks in the open field test. All differences shown were nonsignificant using Fisher’s Least Significant Difference test.

**Supplementary Figure 1.** Neither FMT donors nor FMT recipients show significant behavioral alterations on the open field test. (A) Percent activity in the center of the open field for FMT donor mice withdrawn from morphine and placebo controls. (B) Total activity in the open field for FMT donor mice withdrawn from morphine and placebo controls. (C) Percent activity in the center of the open field for mice who received FMT from morphine withdrawn mice and placebo controls. (D) Total activity in the open field for mice who received FMT from morphine withdrawn mice and placebo controls. ns = nonsignificant using unpaired t-test with Welch’s correction.

**Supplementary Figure 3.** Relative abundance of bacterial taxa significantly altered by morphine withdrawal in female mice. (A) Differentially abundant phylum. (B-H) Differentially abundant genera. (I-J) Differentially abundant species. *p<0.05, **p<0.01, ***p<0.001, ****p<0.0001 using the Mann-Whitney test.

**Supplementary Figure 4.** Relative abundance of bacterial taxa significantly altered by morphine withdrawal in male mice. (A-C) Differentially abundant phyla. (D-K) Differentially abundant genera. (L) Differentially abundant species. *p<0.05, **p<0.01, ***p<0.001 using the Mann-Whitney test.
